# Learning protein-ligand binding affinity with atomic environment vectors

**DOI:** 10.1186/s13321-021-00536-w

**Published:** 2021-08-14

**Authors:** Rocco Meli, Andrew Anighoro, Mike J. Bodkin, Garrett M. Morris, Philip C. Biggin

**Affiliations:** 1 Department of Biochemistry, University of Oxford, Oxford, UK; 2 Department of Statistics, University of Oxford, Oxford, UK; 3 Evotec (UK) Ltd., Abingdon, UK

**Keywords:** Binding affinity, Scoring function, Deep learning

## Abstract

Scoring functions for the prediction of protein-ligand binding affinity have seen renewed interest in recent years when novel machine learning and deep learning methods started to consistently outperform classical scoring functions. Here we explore the use of atomic environment vectors (AEVs) and feed-forward neural networks, the building blocks of several neural network potentials, for the prediction of protein-ligand binding affinity. The AEV-based scoring function, which we term AEScore, is shown to perform as well or better than other state-of-the-art scoring functions on binding affinity prediction, with an RMSE of 1.22 p*K* units and a Pearson’s correlation coefficient of 0.83 for the CASF-2016 benchmark. However, AEScore does not perform as well in docking and virtual screening tasks, for which it has not been explicitly trained. Therefore, we show that the model can be combined with the classical scoring function AutoDock Vina in the context of $\Delta$-learning, where corrections to the AutoDock Vina scoring function are learned instead of the protein-ligand binding affinity itself. Combined with AutoDock Vina, $\Delta$-AEScore has an RMSE of 1.32 p*K* units and a Pearson’s correlation coefficient of 0.80 on the CASF-2016 benchmark, while retaining the docking and screening power of the underlying classical scoring function.

**Supplementary Information:**

The online version contains supplementary material available at 10.1186/s13321-021-00536-w.

## Introduction

Structure-based drug discovery exploits knowledge of protein structures to design novel and potent compounds for a specific target. Protein-ligand docking is one of the main computational tools employed in the early stages of structure-based drug discovery—where more accurate methods, such as free energy calculations [1, 2], are too time-consuming—to predict the binding mode and binding affinity of different ligands in a binding site [3]. The binding mode search is usually guided by a scoring function. Sometimes the scoring function has the dual purposes of finding the binding poses (docking) and predicting the protein-ligand binding affinity (scoring) [4], whilst at other times different scoring functions are used for different purposes (scoring, ranking, docking, or screening).

Scoring functions can be loosely assigned to four classes: physics-based, regression-based, knowledge-based, or machine learning-based [5]. Many scoring functions belonging to the first three categories have been developed over the past decades [6–10]. Despite their successes in reproducing the binding pose, a rapid and accurate prediction of the protein-ligand binding affinity remains a very challenging task [11]. In recent years, machine learning and deep learning scoring functions have consistently improved protein-ligand binding affinity predictions [12]. These improvements build on decades of quantitative structure-activity relationship (QSAR) modelling, where simpler representations and regressors were used [13, 14]. Deep learning architectures—which are outperforming standard algorithms in image recognition and natural language processing [15–19]—are under active research, as demonstrated by the large number of new scoring functions based on deep learning [20–26].

In this work we explore the use of a collection of feed-forward neural networks (NNs), each computing an atomic contribution to the protein-ligand binding affinity. We show that this architecture, combined with atom-centred symmetry functions (ACSFs) to capture the local chemical environment of every atom in the protein-ligand binding site, performs as well as or better than current machine learning and deep learning architectures. This particular representation—commonly employed in the development of neural-network potentials (NNPs) [27, 28]—has the advantage of being translationally and rotationally invariant, unlike NN-based or CNN-based scoring functions that often use an order-dependent input vector or grid-based representations as input.

## Methods

### Atomic environment vectors

In order to predict the binding affinity of a ligand to a target of interest, we need a description of the protein-ligand binding site that allows the key protein-ligand interactions to be learned. Ideally, this representation should depend only on the relative positions of the ligand and the protein—the representation should be invariant under translation, rotation, and mirror operations. However, some machine learning and especially deep learning scoring functions employed in computational drug discovery do not satisfy such conditions: grid-based methods are not translationally or rotationally invariant and need extensive data augmentation [20], while vector-based representations are often order-dependent.

Local representations of the atomic environment satisfying the ideal properties outlined above have been employed with success in quantum machine learning [27, 29–31]. In particular, the ACSFs originally introduced by Behler and Parrinello and further developed to build the Accurate NeurAl networK engINe for Molecular Energies (ANAKIN-ME or “ANI” for short) family of NNPs have been successful in producing accurate molecular properties [27, 28, 32, 33].

Here we employ the ACSFs defined for the ANI family of NNPs in order to represent the protein-ligand binding site, where protein residues with at least one atom within a distance *d* from the ligand are considered.

For each atom *i* of element X in the system, its chemical environment can be represented by combining radial ($G^R_{i;\alpha ,m}$) and angular ($G^A_{i;\alpha ,\beta ,m}$) ACSFs in a one dimensional vector, ${\mathbf {G}}_i^X = \{G_{i;\alpha _1,m_1}^R, \dotsc , G_{i;\alpha _1,\beta _1,m_1}^A, \dotsc \}$—called the atomic environment vector (AEV). *X* corresponds to the element of the atom for which the AEV is being computed, while $\alpha$ and $\beta$ denote the elements of the neighbours within a cutoff radius, $R_c$. The ACSFs capture the atom’s radial and angular chemical environment [28], and their locality is ensured by a cutoff function [27]:

$$\begin{aligned} f_c(R_{ij}) = {\left\{ \begin{array}{ll} \frac{1}{2}\left[ \cos \left( \frac{\pi R_{ij}}{R_c}\right) + 1 \right] \qquad &{} R_{ij} \le R_c \\ 0 \qquad &{} R_{ij} > R_c \end{array}\right. } \end{aligned}$$

Radial symmetry functions are given by [27, 28]:

$$\begin{aligned} G_{i;\alpha ,m}^R = \sum _{\begin{array}{c} j \ne i \\ j\in \alpha \end{array}} e^{-\eta _R (R_{ij} - R_s)^2} f_c(R_{ij}) \end{aligned}$$

where the index *m* runs over the set of parameters $\{\{R_s\}, \{\eta _R\}\}$ and the summation over *j* runs over all the atoms of element $\alpha$; $\eta _R$ controls the width of the radial Gaussian distributions, while $R_s$ controls their radial shift. The angular symmetry function is defined as [28]:

$$\begin{aligned} G_{i;\alpha , \beta , m}^A = 2^{1-\zeta } \sum _{\begin{array}{c} j,k\ne i \\ j\in \alpha , k \in \beta \end{array}} \left[ 1 + \cos \left( \theta _{ijk} - \theta _s \right) \right] ^\zeta e^{-\eta _A \left( \frac{R_{ij} + R_{ik}}{2} - R_s \right) ^2} f_c(R_{ij})f_c(R_{ik}) \end{aligned}$$

where the index *m* runs over the set of parameters $\{\{R_s\}, \{\theta _s\}, \{\eta _A\}, \{\zeta \}\}$ and the summation runs over pairs of atoms of elements $\alpha$ and $\beta$; $\eta _A$ and $R_s$ have the same role of $\eta _R$ and $R_s$ in the radial symmetry function described above, with $\theta _s$ capturing different regions of the angular environment, while $\zeta$ controls the width of the peaks of the ACSF in the angular environment [28].

The AEV ${\mathbf {G}}_i^X$ of atom *i* of element *X*—composed of different ACSFs in a single vector—encodes the neighbour-dependent local atomic environment of atom *i* of element *X*. This corresponds essentially to a fine-grained and flexible atom typing, in contrast to the static and arbitrary atom types employed in standard scoring functions.

**Fig. 1:**
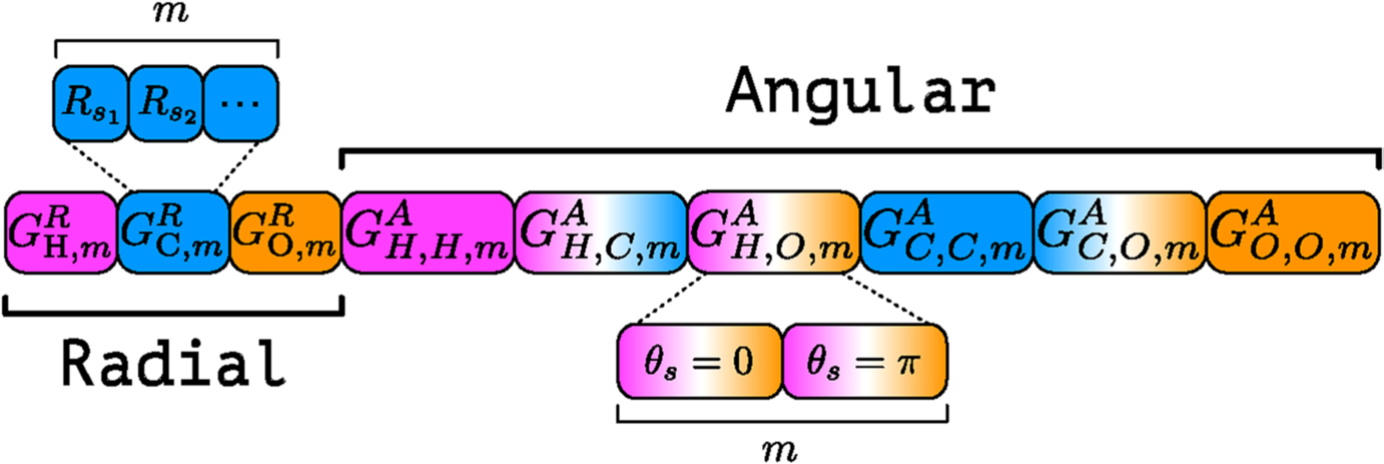
AEV constructed using ACSFs [27, 28] (with $R_s = 0$ and $\{\theta _s\} = \{0,\pi \}$ for angular symmetry functions) for an atom in a system composed only of the elements H, C and O. The radial and angular symmetry functions, $G_{\alpha ,m}^R$ and $G_{\alpha ,\beta ,m}^A$, respectively, are given for the elements $\alpha$ and $\beta$, and iterate over the parameters *m*. Loosely adapted from Gao *et al.* [34]

Figure 1 shows schematically the components of an AEV for an atom in a system composed only of the elements H, C, and O. By construction, this vector is translationally and rotationally invariant as well as invariant under the exchange of two atoms of the same element. An example calculation of ACSFs and AEVs for a simple system is reported in the Supplementary Information for clarity.

In order to keep the size of the AEVs reasonably small, we restrict the parameters of $G_{\alpha ,\beta ,m}^A$ to those of the original Behler-Parrinello formulation: $\{\theta _s\} = \{0,\pi \}$ and $R_s = 0$. All other parameters are the same as those employed in the ANI-1x NNP [28], which results in an AEV size of 200 (for each atom). AEVs are built using the AEVComputer as implemented in TorchANI 2.1 [34].

### Neural network

The NN architecture is implemented using PyTorch 1.7 [35], loosely following the original work of Behler and Parrinello, the ANI family of NNPs, and the TorchANI implementation [27, 28, 34]. It consists of $n_e$ atomic neural networks, where $n_e$ is the number of elements in the dataset. The atomic NNs are standard feed-forward NNs with rectified linear unit (ReLU) activation functions and dropout layers. The outputs of the atomic NNs are then summed together in order to obtain the final estimate of the binding affinity.

**Fig. 2:**
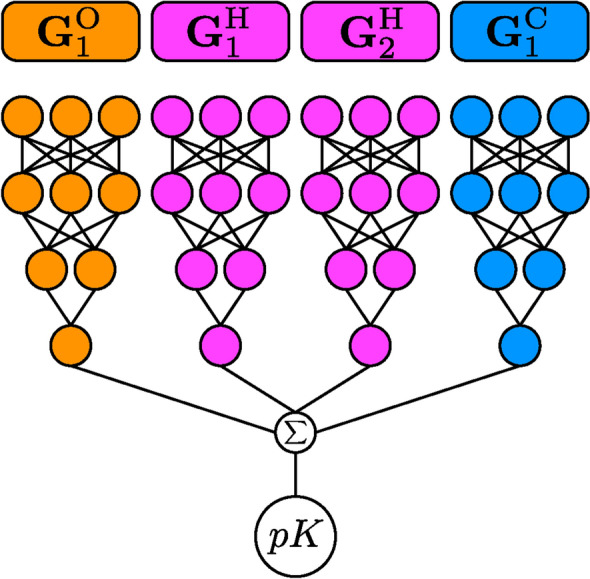
Propagation of AEVs, ${\mathbf {G}}_i^X$, through atomic NNs for the four atoms of a hypothetical system composed of two hydrogen atoms, one carbon atom, and one oxygen atom. The AEVs, ${\mathbf {G}}_i^X$, are constructed for each atom *i* of element *X* as described in the main text and propagated through the atomic NN of the corresponding element (NNs with the same colors have the same weights). All atomic contributions are finally summed together to obtain the p*K* prediction. Loosely adapted from Smith et al. [28]

Figure 2 shows a schematic representation of the model for a hypothetical system composed of two hydrogen atoms, one carbon atom, and one oxygen atom. The AEVs ${\mathbf {G}}^X_i$ corresponding to atoms of the same element *X* are propagated through the same atomic NNs (with the same weights). All atomic contributions are summed together in order to get the final prediction.

The idea behind the decomposition of the binding affinity into atomic contributions is essentially the one that has been proven useful for short-range energy decomposition in NNPs. The negative logarithm of the binding affinity $pK = -\log _{10}(K/c_0)$ is proportional to the Gibbs free energy of binding

$$\begin{aligned} \text {p}K = -\frac{1}{\ln (10)} \frac{\Delta G^\text {bind}_0}{RT} \end{aligned}$$

and therefore decomposing p*K* into atomic contributions corresponds to a decomposition of the Gibbs free energy. As for the total energy in NNPs, this decomposition allows the description of local contributions only [30], but it is very effective in practice—as demonstrated by the success of NNPs in fitting high-dimensional potential energy surfaces [27, 28, 31, 33, 36]. This decomposition also appears to be very effective in generalisation and transferability, since it works for systems much larger than the ones included in the training set [28].

### Training and test datasets

The PDBbind dataset provides protein-ligand complexes with associated experimentally determined inhibition constants, $K_i$, dissociation constants, $K_d$, and $\text {IC}_{50}$ measurements (in decreasing order of preference) [37, 38]. This dataset is divided into two parts: the PDBbind Refined set and the PDBbind General superset. The Refined set only contains high-quality structures with associated $K_i$ or $K_d$ values, while the General set also includes structures with associated $\text {IC}_{50}$ values. A curated subset of the PDBbind Refined set is provided for comparative assessment of scoring functions (CASF) [39, 40].

In this work, the PDBbind 2016 Refined set is used for training and validation while the CASF-2013 and CASF-2016 data sets are used for testing and comparison with other machine learning and deep learning models, as well as classical scoring functions [37–40]. The PDBbind 2016 Refined set is randomly split into training and validation sets with a 90/10 ratio. Systems present in both PDBbind and CASF datasets are removed from the training and validation sets and used only for testing. This procedure ensures that there is no exact overlap (“hard overlap”) of protein-ligand complexes between the PDBbind (training/validation) and CASF (test) datasets, although some overlap with similar targets and ligands remains [39–41]. In order to assess this remaining “soft overlap” between training and test sets—arising from similar proteins, similar binding sites, and similar ligands—we use the subset of the PDBbind 2016 dataset proposed by Su et al. [41].

A detailed analysis of the CASF-2013 and CASF-2016 test sets—including the distribution of the protein-ligand binding constants and of some key properties of the protein-ligand complexes—is reported by Li et al. [39] (CASF-2013) and Su et al. [40] (CASF-2016). In particular, the CASF-2016 dataset is composed of 57 protein classes each containing 5 protein-ligand complexes—with at least 90% sequence similarity [40]. The CASF-2013 dataset is smaller in size, with 65 protein classes each containing 3 protein-ligand complexes [39].

Ligand SDF or MOL2 files from the datasets were either converted to PDB files using OpenBabel [42] and parsed using MDAnalysis (for scoring and ranking) or parsed directly with OpenBabel’s Python bindings (docking and screening) [43–45]. Protein PDB files were discarded when the element column was absent or could not be parsed correctly by MDAnalysis (this never occurred for the test set). All water molecules were removed from the dataset. All the systems in the PDBbind and CASF dataset were automatically protonated using OpenBabel [42], and given the size of the dataset the protonation state was not further assessed.

The complexity of the NN model grows quickly with the number of atomic species present in the dataset since every element requires its own atomic NN. For this reason, we adopted two different strategies to deal with metal centres: selecting only protein and ligand atoms (retaining protein residues with at least one atom within distance *d* from the ligand and discarding all metal centers), or selecting protein and ligand atoms (retaining protein residues with at least one atom within distance *d* from the ligand) and mapping metal centers to a single dummy atom. Additionally, we removed the few selenoproteins present in the training or validation sets. When selecting only protein and ligand atoms, the following elements remained (in order of abundance for the ligands, see Additional file 1: Figure S3): H, C, O, N, S, P, F, Cl, Br, I. This resulted in a total of 10 atomic NNs, one for each element. When metal centers were kept (see Additional file 1: Figure S4), all atoms outside of the previous list were mapped to a dummy element, X.

When “hard overlaps” with CASF-2016 were removed, the final training set consisted of 3377 complexes while the validation set consisted of 376 complexes. When “hard overlaps” with CASF-2013 were removed, the final training set consisted of 3464 complexes while the validation set consisted of 385 complexes. The CASF test sets are left unchanged.

Protein-ligand complexes 4O3C and 4IGT were removed from the PDBbind Refined Set since they contain lithium, which is not supported by AutoDock Vina [10], the classical scoring function used as baseline in this work.

The advantage of mapping metal centers to a dummy atom is that metalloproteins, which are notoriously difficult to treat with docking and classical molecular dynamics [46, 47], are supported by our method. However, our treatment has the drawback of considering all metal atoms as equivalent, irrespective of their coordination number. As more experimental data on metalloproteins becomes available, more elements could be added to the model (with an increased computational cost).

### $\Delta$-learning

$\Delta$-learning is a powerful machine learning approach where the model is trained to predict the corrections to a baseline towards the target value, instead of predicting the target value itself [48]. This approach has been applied successfully to the prediction of molecular properties from quantum mechanical calculations as well as for binding affinity predictions [48–50]. In the context of docking scoring functions, a $\Delta$-learning approach has the advantage of retaining the good docking power of traditional methods while significantly improving the scoring function [49].

In this work we explored the use of a $\Delta$-learning approach in combination with the AutoDock Vina scoring function [10]. The $\Delta$-AEScore scoring function is therefore given by:

$$\begin{aligned} \Delta \text {-AEScore} = S + \Delta \end{aligned}$$

where *S* is the standard AutoDock Vina score (in p*K *units) and $\Delta$ is the learned correction.

### Consensus scoring

In order to compensate for the variability introduced by random weights initialization and stochastic optimization, we investigated the use of consensus scoring in order to evaluate our models. Consensus scoring has been shown, in some cases, to improve performance across targets in structure-based virtual screening [51, 52].

During training, a total of five models were randomly initialized and independently trained. Final predictions were obtained as the average protein-ligand binding affinity of the models. This technique also allows the computation of the standard deviation associated with each prediction. The benefits of consensus scoring are analysed retrospectively below.

### Software

Our implementation is based on open source software from the Python [53] ecosystem. This includes: TorchANI 2.1 [34], PyTorch 1.7 [35], MDAnalysis 2.0-dev [43, 44], OpenBabel 3.1 [45, 54], NumPy 1.19 [55], SciPy 1.5 [56], pandas 1.1 [57] , Matplotlib 3.3 [58], seaborn 0.11 [59], scikit-learn 0.23 [60], and pytest 6.0 [61].

## Results

### AEScore

#### Hyperparameters optimization

The hyperparameters of our model—the number and size of layers in the elemental NNs, dropout probability, batch size, and protein-ligand distance, *d*—were optimized with a grid-based method and manually fine-tuned in order to maximize the Pearson’s correlation coefficient between the predicted and experimental binding affinities on the validation set.

We found that a protein-ligand distance $d=3.5$ Å and 256-128-64-1 feed-forward NNs performed best when combined with a batch size of 64 and a dropout probability of 25%.

Additional file 1: Table S2 shows the performance of the model—with consensus scoring—on the validation test for different values of *d*. Using a distance of $d=4.0$ Å does not change the performance, compared to $d=3.5$ Å. However, the larger number of protein atoms causes the computational time to be increased. Visual inspection of a selection of systems showed that the $d=3.5$ Å selects the important residues in the binding site.

The model’s weights are optimized using the ADAM optimizer with a learning rate of $1 \times 10^{-4}$ and using PyTorch’s default parameters, $\beta _1 = 0.9$ and $\beta _2=0.999$ [35, 62].

Dropout layers are usually not employed in NNPs, but our hyperparameter search shows that they increase the performance of our model by decreasing overfitting on the training set, thus improving transferability.

#### Scoring power

The scoring power of a scoring function measures the linear correlation between predicted and experimental binding affinities and it is usually quantified by Pearson’s correlation coefficient:

$$\begin{aligned} r = \frac{\sum _i ({\hat{y}}_i - \langle {\hat{y}}\rangle ) (y_i - \langle y\rangle )}{\sqrt{\sum _i ({\hat{y}}_i - \langle {\hat{y}}\rangle )^2} \sqrt{\sum _i (y_i - \langle y\rangle )^2}} \end{aligned}$$

where *y* denotes experimental values, ${\hat{y}}$ denotes predicted values, and $\langle \cdot \rangle$ denotes the average over all experimental or predicted values.

**Fig. 3:**
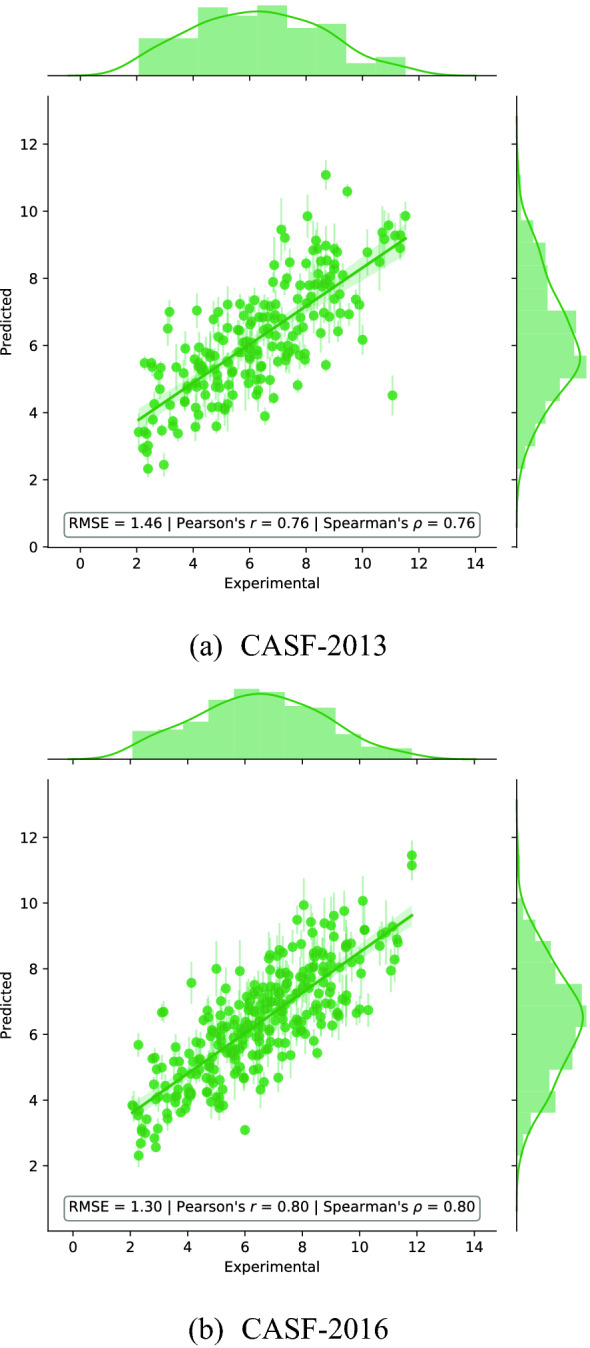
Predicted versus experimental binding affinityfor AEScore, expressed in p*K* units, when only protein and ligand atoms are retained

Figure 3 shows the predictions of our model versus the experimental values of the binding affinity for the CASF-2013 and CASF-2016 benchmark data sets—when only protein and ligand atoms are considered. Our model achieves an RMSE of 1.30 p*K* units and a Pearson’s correlation coefficient of 0.80 on the CASF-2016 test set, and an RMSE of 1.46 p*K* units and a Pearson’s correlation coefficient of 0.76 on the CASF-2013 test set. Error bars show the standard deviation of the predictions obtained with consensus scoring (average over five independently trained models).

Confidence intervals (CIs) for the correlation coefficient can be obtained by bootstrapping (with 10000 bootstrap replicates), as described in the CASF evaluation [40]. The 90% CI for the Pearson’s correlation coefficient for the CASF-2016 test set is $[0.76, 0.83]_{\text {CI 90}\%}$, while for the CASF-2013 test set it is $[0.68, 0.81]_{\text {CI 90}\%}$.

**Fig. 4:**
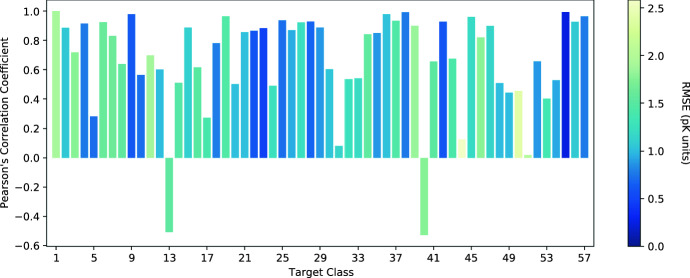
Per-class Pearson’s correlation coefficient, with each bar color-coded by the corresponding RMSE in p*K* units, for the 57 classes of the CASF-2016 dataset

Figure 4 shows a breakdown of the Pearson’s correlation coefficient (and the RMSE) for each protein class in the CASF-2016 benchmark data set. We see that the performance of AEScore is class-dependent and there is no clear correlation between the Pearson’s correlation coefficient and the RMSE (by comparing class #1 and class #55, for example). For the majority of targets, the predicted binding affinity is well correlated with the corresponding experimental value. Only a few classes have a low correlation coefficient and two classes show negative correlation. The classes with negative correlation are (refer to the supplementary information of Su et al. [40] for the full list of classes): $\beta$-lactoglobulin (class 13) and queuine tRNA-ribosyltransferase (class 40). The average and median Pearson’s correlation coefficients across all target classes are 0.67 and 0.82, respectively.

Additional file 1: Figure S5 compares per-class Pearson’s correlation coefficient obtained with AEScore (and reported in Fig. 4) using results obtained with GNINA [20, 21], a CNN-based scoring function. We see that for most classes the Pearson’s correlation coefficient obtained with both methods is similar. However, there are some classes where the difference between the two methods is larger than 0.2 and in such cases GNINA shows a better correlation in most cases (15 out of 21).

This protein class-dependence opens up the scope for protein-specific models or fine-tuning (for example using transfer learning) which are likely to improve per-class performance [63].

#### Consensus scoring

In the previous section we employed consensus scoring—with five independently trained models—since this has previously been shown to improve performance [51, 52]. A small performance boost is also obtained in our case, as it can be verified retrospectively.

If we consider the CASF-2016 dataset, the average correlation coefficient of the five independent models is 0.77 (minimum 0.77, maximum 0.78) while consensus scoring reaches 0.80—better than the best-performing individual model amongst the five. The same observation is true for the RMSE on the same test set. The average RMSE is 1.38 p*K* units (minimum 1.35, maximum 1.42) while the consensus scoring has a RMSE of 1.30 p*K* units—which is lower than the best-performing model amongst the five.

#### Implicit hydrogen atoms

To assess the impact of automatic protonation using OpenBabel [54] we also trained AEScore without hydrogen atoms for both the protein and the ligand. This results in the removal of one atomic NN, thus decreasing the number of parameters in the model.

Training the model without hydrogen atoms does not seem to consistently affect the performance of our model: we observe a small decrease in performance with the CASF-2013 test set and a small gain with the CASF-2016 test set. For the CASF-2013 test set, we obtain a Pearson’s correlation coefficient of $0.75 \in [0.69, 0.80]_{\text {CI 90}\%}$ and an RMSE of 1.48 p*K* units while for the CASF-2016 test set we obtain a Pearson’s correlation coefficient of $0.81 \in [0.77, 0.84]_{\text {CI 90}\%}$ and an RMSE of 1.28 p*K* units.

Per-class Pearson’s correlation coefficient (and RMSE) for the CASF-2016 test set for the model trained without hydrogen atoms is shown in Additional file 1: Figure S6. Again, there is no clear relationship between Pearson’s correlation coefficient and RMSE. In this case, the average Pearson’s correlation coefficient is 0.69 while the median is 0.85.

#### Metalloproteins

When metal centers are included, they are mapped to a dummy element X. As we can see from Additional file 1: Figure S4, Zn is the most abundant metal center in our dataset (545 systems), followed by Mg (142 systems). All other metal centers appear in fewer than 60 systems.

With the metal centers mapped to a dummy element X, we obtain a Pearson’s correlation coefficient of $0.80 \in [0.76, 0.83]_{\text {CI 90}\%}$ and an RMSE of 1.31 p*K* units on the CASF-2016 benchmark. When hydrogen atoms are removed, we find a Pearson’s correlation coefficient of $0.81 \in [0.77, 0.84]_{\text {CI 90}\%}$ and a RMSE of 1.31 p*K* units.

#### Similarity between training and test sets

As mentioned above, we removed the systems appearing in the CASF-2016 and CASF-2013 benchmark datasets from the training sets (removing the so-called “hard overlap”). However, some “soft overlap”—arising from similar proteins, similar binding sites, and similar ligands—between the training and test sets remains and could therefore artificially inflate the results. This is a known problem as shown by Boyles et al. [4] and, more recently, by Su et al. [41] who both proposed non-redundant subsets of the PDBbind refined set with decreasing similarity with respect to the CASF-2016 test set. Such non-redundant datasets allow assessing how scoring functions behave when the “soft overlap” between the training and test sets is incrementally reduced.

In the work of Su et al. [41] the similarity between the training and test sets is measured by three metrics: similarity between protein sequences, similarity between ligand shapes, and similarity between binding pockets. If two protein-ligand complexes—one in the training set, the other in the test set—have all three similarity metrics above a given threshold they are considered redundant. All redundant complexes are removed from the training set with an iterative procedure until the remaining complexes form a representative, non-redundant training set for the given similarity threshold [41].

**Fig. 5:**
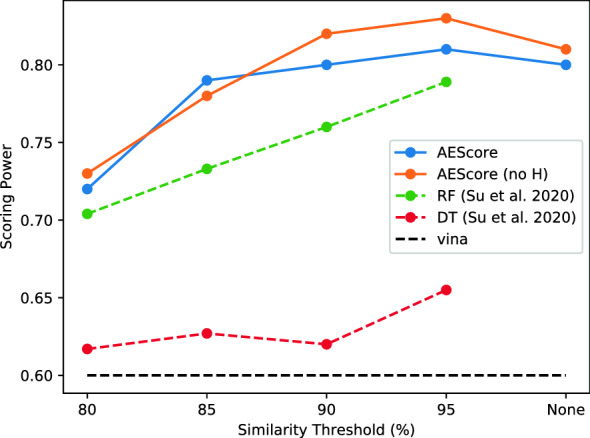
Scoring power of AEScore (with and without hydrogen atoms) as a function of the similarity threshold between the training and test sets, as defined by Su et al. [41]. The raw data for the RF and DT scoring functions was kindly provided by Su et al. [41] upon request. RF and DT are respectively the best and worst performing models (at the 95% similarity threshold) presented in Su et al. [41] and are consistently outperformed by AEScore

Figure 5 shows the performance of our model on the CASF-2016 dataset when trained on the non-redundant training sets proposed by Su et al. [41], with different similarity thresholds (“None” indicates that only the “hard overlap” between training and test sets is removed). We see that as the overlap threshold between the training and test sets increases, the performance of our model also increases. Interestingly, a similarity threshold of 95% does not negatively affect our scoring function, in contrast with other machine learning scoring functions [41]. This trend is similar to the RF model of Su et al. [41], which is consistently outperformed by our model. Other machine learning scoring functions evaluated by Su et al. [41] are effectively negatively affected by removing structurally redundant samples already at high thresholds.

We also found that the model with a similarity threshold of 95% (denoted AEScore_95_ hereafter) seems to perform slightly better than the model trained by only removing the “hard overlap”. This could be attributed to the removal of some inconsistencies in the training set, introduced by experimental errors, or simply to the variability of the training procedure (minibatches, dropouts, etc.). The AEScore_95_ model is our best performing model on the CASF-2016 test set (Pearson’s correlation coefficient of $0.83 \in [0.79, 0.86]_{\text {CI 90}\%}$, RMSE of 1.22 p*K* units) and it performs very well compared to other state-of-the-art scoring functions (see discussion of Figure 10)—although differences with other top-performing methods might not be statistically significant.

#### Ranking power

The ranking power of a scoring function measures its ability to rank different ligands—in a given binding pose—according to their binding affinity against a particular target. The ranking power is usually measured by three quantities: Spearman’s (rank-)correlation coefficient, Kendall’s (rank-)correlation coefficient and the predictive index (PI) [40, 64].

Our scoring function AEScore has an average Spearman’s correlation coefficient of $0.64 \in [0.54, 0.71]_{\text {CI 90}\%}$. This is similar to the best classical scoring function evaluated in the CASF-2016 [40], although it is within the 90% confidence interval. The same observation remains true for the average Kendall’s correlation coefficient of $0.55 \in [0.47, 0.62]_{\text {CI } 90\%}$ and for the PI of $0.67 \in [0.58, 0.73]_{\text {CI 90}\%}$.

Interestingly, if hydrogen atoms are removed the ranking power does not change. When hydrogen atoms are ignored, the Spearman’s correlation coefficient becomes $0.63 \in [0.54, 0.71]_{\text {CI 90}\%}$, the Kendall’s correlation coefficient becomes $0.56 \in [0.48, 0.63]_{\text {CI 90}\%}$, and the PI becomes $0.66 \in [0.57, 0.74]_{\text {CI 90}\%}$.

**Fig. 6:**
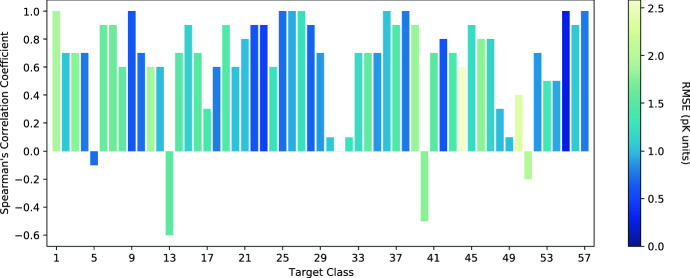
Per-class Spearman’s correlation coefficient, with each bar color-coded by the corresponding RMSE in p*K* units, for the 57 classes of the CASF-2016 dataset

Figure 6 shows the per-class Spearman’s rank-correlation coefficient, while the per-class Kendall’s correlation coefficient is reported in Additional file 1: Figure S7. For Spearman’s correlation coefficient we now have four classes with negative correlation. Classes 13 and 40 ($\beta$-lactoglobulin and queuine tRNA-ribosyltransferase, respectively) also had a negative Pearson’s correlation coefficient, while classes 5 (alpha-L-fucosidase) and 51 (transporter) did not. For Kendall’s correlation coefficient we have only three classes with negative correlation: classes 13, 40, and 51. A few other classes have no correlation.

#### Docking power

AEScore has been developed with the intent of predicting the binding affinity of a given protein-ligand complex. However, scoring functions can also be used to determine correct binding poses. Therefore we evaluate the docking power of AEScore using the docking decoys provided in CASF-2016 dataset [40].

If we consider a correct binding pose as one with a root mean squared deviation (RMSD) from the crystallographic binding mode that is smaller than 2 Å, we can define the docking success rate as the percentage of targets with a good pose ranked amongst the top one, top two or top three poses.

AEScore has a success rate of $35.8\% \in [30.9, 40.4\%]_{90\% \text { CI}}$ for the top one pose, a success rate of $54.4\% \in [48.8, 58.6\%]_{90\% \text {CI}}$ for the top two poses and a success rate of $60.4\% \in [54.7, 64.2\%]_{90\% \text { CI}}$ for the top three poses. Such low success rates are comparable with the worst classical scoring functions evaluated on the CASF-2016 benchmark [40]. This low success rate is also observed with other deep learning scoring functions: a recent pre-print study presenting a CNN-based scoring function, AK-score [65], reports a top one success rate of 34.9 (single) or 36.0% (ensemble) [65].

These results are not surprising, since AEScore has been trained to predict the experimental binding affinity given a protein-ligand complex and has therefore never been exposed to high-RMSD binding poses (decoys). In order to use the scoring function to determine low-RMSD poses one has to train for such task. One way to train a scoring function for docking is to train a pose classifier (distinguishing low RMSD poses from high RMSD poses) [20], but this requires a change in the model architecture. Another way to tailor a machine learning scoring function for docking is to train on docking scores as done for AGL-Score [66]. A third way to improve binding affinity predictions while retaining the good docking and screening power of some classical scoring functions is to use $\Delta$-learning [49]. In this work we explore the latter approach.

### $\Delta$-AEScore

#### $\Delta$-learning with AutoDock Vina

The use of AEVs combined with a collection of feed-forward NNs has proven successful to predict protein-ligand binding affinities on the CASF-2013 and CASF-2016 benchmark datasets using exclusively elements and atomic coordinates, as demonstrated above. Unfortunately, the results of the docking power test were unexpectedly deceiving. However, it has been previously demonstrated that a $\Delta$-learning approach can retain the good screening power of a scoring function while improving the performance in the docking and screening power tests [49].

In the $\Delta$-learning approach, a classical scoring function is used to obtain a crude prediction of the binding affinity, which is subsequently corrected with a machine learning or deep learning scoring function. If corrections to the AutoDock Vina scoring function can be learned by our model, combining such corrections with the docking power of AutoDock Vina would provide a scoring function with both good scoring and docking powers [49].

In order to combine AutoDock Vina and the experimental data of PDBbind, AutoDock Vina scores, *S*, are converted to p*K* values using

$$\begin{aligned} pK = -\log _{10} \left( e^{\frac{S}{RT}} \right) , \end{aligned}$$

where $T={295}\,{\mathrm{K}}$ and *R* is the ideal gas constant.

#### Scoring power

**Fig. 7:**
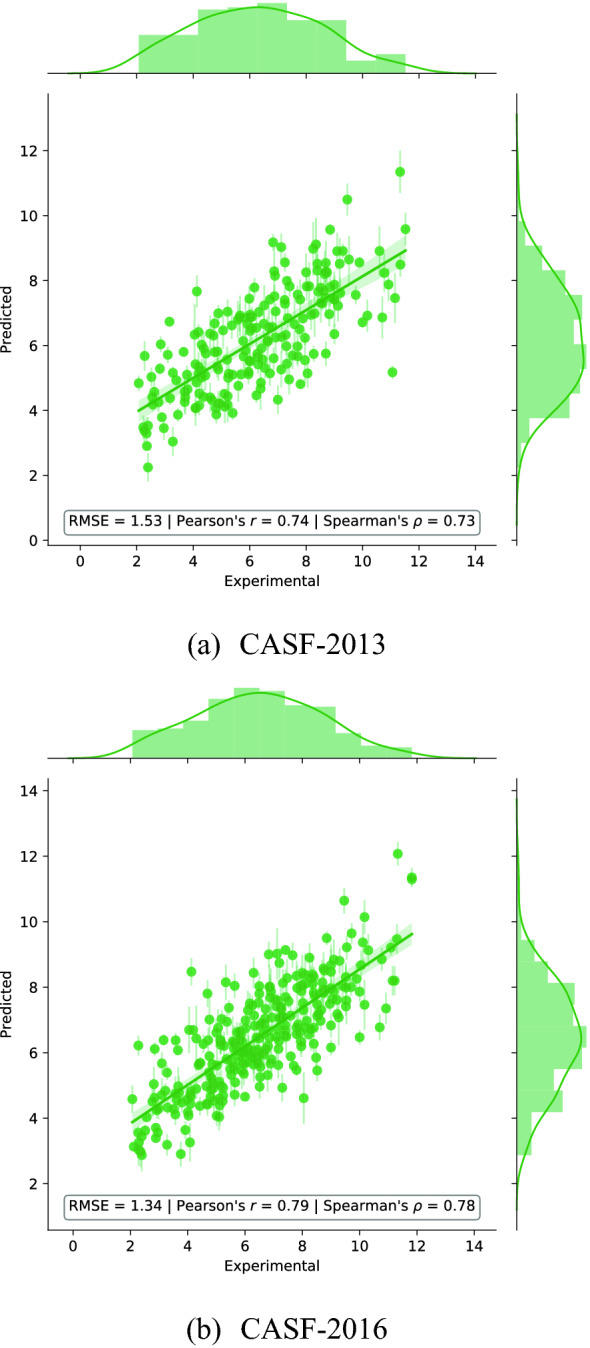
Predicted versus experimental binding affinity using the $\Delta$-learning approach with $\Delta$-AEScore, expressed in p*K* units, when only protein and ligand atoms are retained

Figure 7 shows the predictions of our model versus the experimental values of the binding affinity for the CASF-2013 and CASF-2016 benchmark data sets. $\Delta$-AEScore achieves an RMSE of 1.53 p*K* units and a Pearson’s correlation coefficient of $0.74 \in [0.67, 0.78]_{\text {CI 90}\%}$ on the CASF-2013 test set and an RMSE of 1.34 p*K* units and a Pearson’s correlation coefficient of $0.79 \in [0.75, 0.82]_{\text {CI 90}\%}$ on the CASF-2016 test set. The performance is slightly worse than that of AEScore, indicating that corrections to the AutoDock Vina native scoring function are also difficult to learn. This is probably caused by the approximate nature of classical scoring functions.

Table 1 compares our $\Delta$-learning results on the CASF-2013 and CASF-2016 data sets with the $\Delta _\text {vina}\text {RF}$ scoring function, arguably the most successful implementation of this approach [49]. Our model performs significantly better than $\Delta _\text {vina}\text {RF}$ on the CASF-2013 dataset and comparably on the CASF-2016. It is worth noting that $\Delta _\text {vina}\text {RF}$ is the best scoring function on the scoring and ranks power tests for the CASF-2016 benchmark, and is ranking consistently amongst the top scoring functions for the docking and screening power tests. However, $\Delta _\text {vina}\text {RF}$ is calibrated on protein-ligand complexes from the PDBbind, which overlaps with $\sim$50% of the CASF-2016 test set and its performance might therefore have been artificially enhanced by a large overlap between the training and test sets [40].

Both $\Delta _\text {vina}\text {RF}$ and $\Delta$-AEScore outperform the classical scoring function AutoDock Vina in the scoring power test, by a large margin [40].

**Table 1:** Performance of $\Delta$-AEScore compared to the $\Delta _\text {vina}\text {RF}$ for affinity prediction on the CASF-2013 and CASF-2016 benchmarks. For $\Delta$-AEScore the “hard overlap” between the training and both test sets is removed while for $\Delta _\text {vina}\text {RF}$ only the “hard overlap” between the training set and CASF-2013 is removed [49, 67]. The best performance for each test set is underlined. RMSE values are given in p*K* units

Model	Training set	Test set	RMSE	Pearson’s *r*
$\Delta$-AEScore$^{\dag }$	Refined 2013	CASF-2013	1.53	0.74
$\Delta$-AEScore$^{\dag }$ (no H)	Refined 2013	CASF-2013	1.52	0.74
$\Delta _\text {vina}\text {RF}$ [49]	Refined 2013	CASF 2013	—	0.69
Vina (optim)	—	CASF-2013	1.82	0.61
$\Delta$-AEScore$^{\dag }$	Refined 2016	CASF-2016	1.34	0.79
$\Delta$-AEScore$^{\dag }$ (no H)	Refined 2016	CASF-2016	1.32	0.80
$\Delta _\text {vina}\text {RF}$ [40, 49]	Refined 2013	CASF 2016	—	0.81
Vina (optim)	—	CASF-2016	1.75	0.59

$$^\dag$$ This work

#### Ranking power

In terms of ranking power $\Delta$-AEScore has a Spearman’s correlation coefficient of $0.59 \in [0.47, 0.68]_{90\% \text { CI}}$, a Kendall’s correlation coefficient of $0.52 \in [0.42, 0.60]_{90\% \text { CI}}$ and a PI of $0.61 \in [0.49, 0.69]_{90\% \text { CI}}$ on the CASF-2016 benchmark. For the CASF-2013 benchmark, $\Delta$-AEScore has a Spearman’s correlation coefficient of $0.61 \in [0.47, 0.71]_{90\% \text { CI}}$, a Kendall’s correlation coefficient of $0.58 \in [0.44, 0.67]_{90\% \text { CI}}$ and a PI of $0.63 \in [0.49, 0.73]_{90\% \text { CI}}$.

The performance of $\Delta$-AEScore in the ranking power test is lower than the performance of AEScore. This is to be attributed to the poor performance of AutoDock Vina on this benchmark, with a Spearman’s correlation coefficient of $0.53 \in [0.43, 0.61]_{90\% \text { CI}}$ on the CASF-2016 benchmark [40]. However, the use of AEScore on top of AutoDock Vina allows us to improve the performance of the latter in both scoring and ranking.

#### Docking power

We next wanted to see if the corrections to the AutoDock Vina scoring function can be applied in the context of docking. Using the docking decoys of the CASF-2016 benchmark dataset we obtain a top one success rate of $85.6\% \in [81.1, 88.1\%]_{90\% \text { CI}}$, a top two success rate of $94.4\% \in [90.9, 95.8\%]_{90\% \text { CI}}$ and a top three success rate of $95.8\% \in [92.6, 96.8\%]_{90\% \text { CI}}$. This is a very significant improvement on the previous results obtained with AEScore.

The top one performance is lower than Autodock Vina itself, which performs extremely well in this benchmark with a top 1 success rate of $90.2\% \in [86.7, 92.6\%]_{90\% \text { CI}}$ (when the native ligand binding pose is included), and compared to the performance of $\Delta _\text {vina}\text {RF}$, the second-best performing scoring function in CASF-2016 with a top 1 success rate of $89.1\% \in [85.6, 91.6\%]_{90\% \text { CI}}$ [40]. However, the much higher performance compared to AEScore indicates that the protein-ligand binding site representation and the model architecture used for AEScore are amenable to $\Delta$-learning. We thus have good scoring power—significantly better than AutoDock Vina alone—while retaining the excellent docking power of Autodock Vina.

#### Screening power

Given the good success rate of $\Delta$-AEScore in the docking power test, we wanted to evaluate $\Delta$-AEScore in the context of virtual screening as well. The screening power test assesses the ability of a scoring function to identify true binders among a large pool of decoys. There are two types of screening power tests provided in the CASF-2016 benchmark: in forward screening, the goal is to identify the true binders for a given target, while in reverse screening, the goal is to identify a potential target for a given active compound [40].

For the forward screening power test, $\Delta$-AEScore ranks the best ligand among the top 1% of candidates with a success rate of $19.3\% \in [10.5, 26.3\%]_{90\% \text { CI}}$. The top 5% success rate and the 10% success rates are $49.1\% \in [36.8, 57.9\%]_{90\% \text { CI}}$ and $54.4\% \in [42.1, 63.2\%]_{90\% \text { CI}}$, respectively. The top 1% success rate is rather low compared to Autodock Vina ($29.8\% \in [19.3, 38.6\%]_{90\% \text { CI}}$) and $\Delta _\text {vina}\text {RF}$ ($42.1\% \in [29.8, 50.9\%]_{90\% \text { CI}}$), but top 5% and top 10% performances are in line with $\Delta _\text {vina}\text {RF}$ and better than AutoDock Vina itself [40]. Again, it is worth re-iterating that the reported performance of $\Delta _\text {vina}\text {RF}$ on CASF-2016 might be artificially inflated by the overlap between training and test sets [40].

Another quantitative metric of the screening power is the enrichment factor (EF), defined by:

$$\begin{aligned} \text {EF}_\alpha = \frac{\text {TB}_\alpha }{\alpha \text {TB}_\text {tot}} \end{aligned}$$

where $\text {TB}_{\alpha }$ denotes the number of true binders amongst the top $\alpha \%$ candidates and $\text {TB}_\text {tot}$ is the total number of true binders. $\Delta$-AEScore has an average $\text {EF}_{1\%}$ of $6.16 \in [4.14, 8.75]_{90\% \text { CI}}$, an average $\text {EF}_{5\%}$ of $3.76 \in [2.94, 4.63]_{90\% \text { CI}}$ and an average $\text {EF}_{10\%}$ of $2.48 \in [2.02, 3.00]_{90\% \text { CI}}$. The EF are not too far from AutoDock Vina’s EF on CASF-2016, with an $\text {EF}_{1\%}$ of $7.7 \in [5.37, 10.97]_{90\% \text { CI}}$ [40]. $\Delta _\text {vina}\text {RF}$ is again amongst the top performing scoring functions on CASF-2016, not withstanding the training/testing caveats discussed above; $\Delta _\text {vina}\text {RF}$ $\text {EF}_{1\%}$ is $11.73 \in [8.84,15.41]_{90\% \text { CI}}$ [40].

For reverse screening on the CASF-2016 benchmark, we obtain a top 1% success rate of $11.9\% \in [8.8\%, 15.1\%]_{90\% \text { CI}}$, a top 5% success rate of $19.3\% \in [15.4\%, 23.2\%]_{90\% \text { CI}}$ and a top 10% success rate of $27.0\% \in [22.5\%, 30.9\%]_{90\% \text { CI}}$. Again, the results are similar to AutoDock Vina ($13.7\% \in [10.5\%, 16.8\%]_{90\% \text { CI}}$) and slightly worse than the optimistic values reported for $\Delta _\text {vina}\text {RF}$ ($15.1\% \in [11.6\%, 18.6\%]_{90\% \text { CI}}$) [40].

### Ligand-only affinity prediction

To test the effect of protein information in the binding affinity prediction and to elucidate possible biases in the dataset [68], we also trained a model with only the ligand atoms ($d = 0$ Å). The AEVs’ parameters used to describe ligand atoms are left unchanged.

**Fig. 8:**
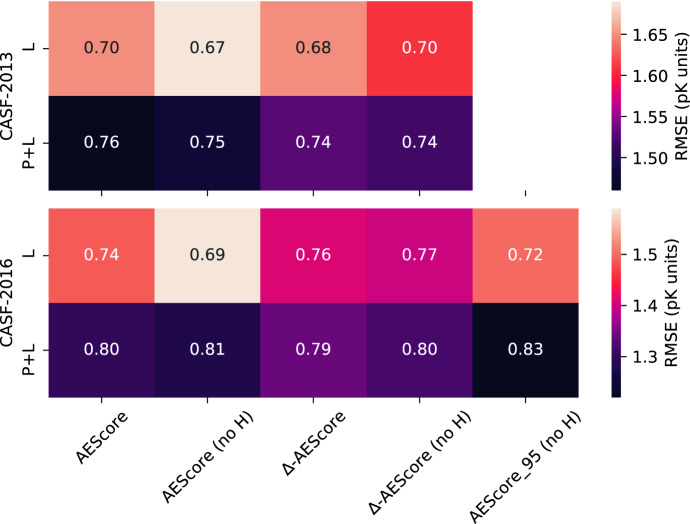
Pearson’s correlation coefficient for different models incorporating atoms from the protein and the ligand (P + L, $d = {3.5}$ Å) or atoms of the ligand only (L), for the CASF-2013 and CASF-2016 benchmarks. Each box is color-coded by the corresponding RMSE in p*K* units

For the CASF-2013 dataset we obtained an RMSE of 1.65 p*K* units and a Pearson’s correlation of 0.70, while for the CASF-2016 dataset we obtained an RMSE of 1.49 p*K* units and a Pearson’s correlation of 0.74 (when only protein and ligand atoms are kept and systems are automatically protonated). Figure 8 also reports the results when hydrogen atoms are removed and when the model is trained on a dataset with a protein/ligand/pocket similarity threshold of 95% similarity with the training set.

As shown in Fig. 8 (and, equivalently, in Additional file 1: Table S3; Figure S10), the performance of the model in absence of protein atoms (L) is always worse than that obtained when including both ligand and protein atoms (P + L). This indicates that the model is able to exploit the additional information about the binding site provided by the protein atoms to improve binding affinity predictions. However, the difference is not as striking as one might expect.

The same observations apply to the $\Delta$-learning approach, although the difference between protein-ligand (P + L) and ligand-only (L) models is even less pronounced. This suggests that corrections to the AutoDock Vina scoring function mainly stem from the information about the ligand and that information about the protein target plays a minor role.

The fact that AEScore models using only information about the ligand already perform well is in line with recent work from Boyles et al. [4] who showed that ligand features alone are predictive of the mean protein-ligand binding affinity in PDBbind [4]. Additionally, ligand information plays a significant role in affinity prediction in deep learning models as well [52, 69, 70]. For ligand-only predictions, AEScore is essentially learning a conformation-dependent fingerprint of the active ligand and using such information to predict the mean binding affinity of said ligand; RDKit descriptors alone, combined with a random forest model, can already achieve a Pearson’s correlation coefficient of 0.71 on CASF-2013 and of 0.76 on CASF-2016, as demonstrated by Boyles et al. [4]. Our results suggest that the AEScore model presented here can use AEVs as 3D ligand fingerprints and use such information to predict the average binding affinity of a ligand in the same way RDKit descriptors allow.

Work parallel to ours recently investigated the application of Smooth Overlap of Atomic Positions (SOAP) [30]—another widely used and related structural representation for molecules and materials [71]—for 3D QSAR [72]. The method is shown to perform competitively with fingerprint-based methods as well as state-of-the-art graph neural networks.

### Visualization

One advantage of working with atomic coordinates directly and using an end-to-end differentiable model is that the gradient of the output (and, eventually, of the loss function) can be computed with respect to the atomic coordinates. This technique has been previously used to interpret CNN-based scoring functions [73]: the gradient of the output with respect to the atomic coordinates indicates where the model would like the atoms to “move” to optimise (improve) the binding affinity (see SI for details).

**Fig. 9:**
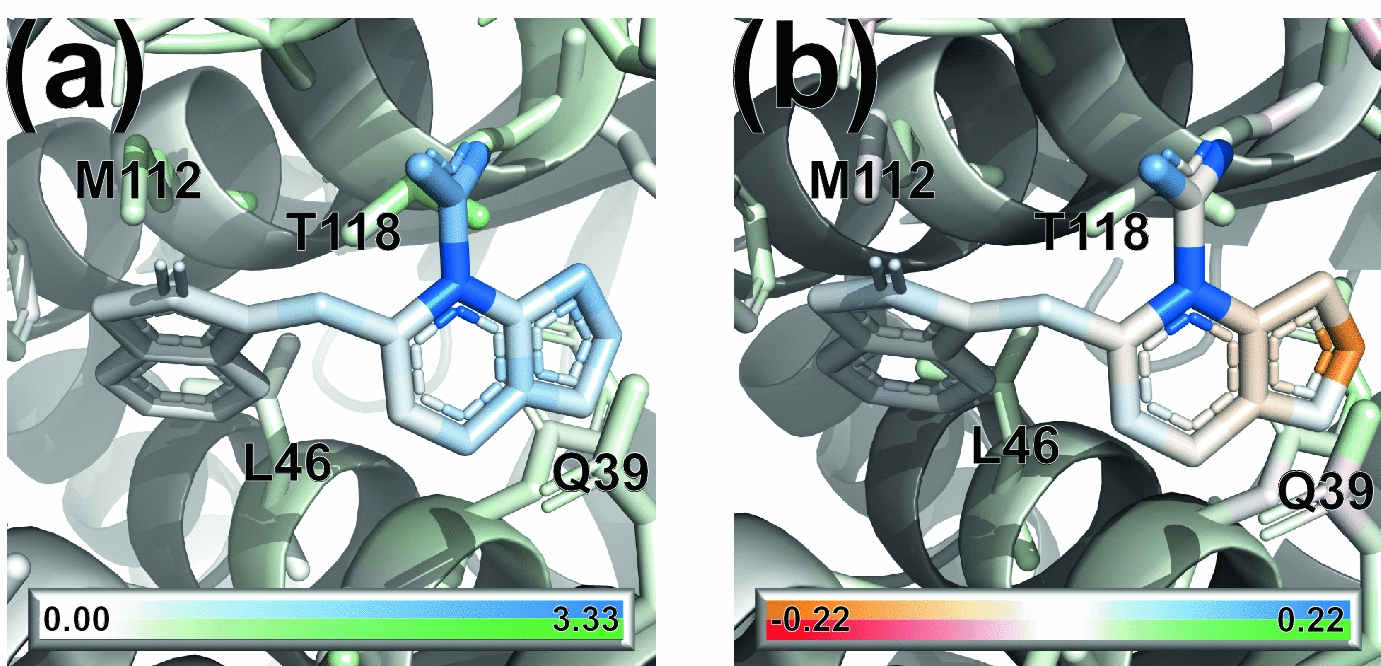
Visualization of** a** the norm of the gradient of the predicted binding affinity with respect to atomic coordinates, and** b** the atomic contributions to the total binding affinity for a small molecule inhibitor bound to HIV type 1 integrase (PDB ID 3ZT2). Ligand contributions go from orange (negative) to blue (positive) while protein contributions go from red (negative) to green (positive); white represents the zero

Figure 9a shows the magnitude of the gradients for ligand and protein atoms for the complexes of the CASF-2016 test set with the lowest absolute error (PDB ID 3ZT2): the gradients are small everywhere, with the exception of a particular functional group of the ligand.

In future iterations of the model, the gradients of the output with respect to the atomic coordinates could be employed as fictitious “forces” for a local geometry optimisation: atoms can be displaced along the gradient with standard optimisation techniques in order to obtain new configurations that optimise (increase) the binding affinity [74].

Since the model prediction comes from atomic contributions, it is interesting to visualize such contributions as well. To compute atomic contributions, a single evaluation of the protein-ligand binding affinity is required. This is in contrast with the use of masking for non-additive models, where a forward pass is needed after removing, in turn, each ligand atom and residue in the binding site, at a greater computational expense [73]. Figure 9(b) shows the atomic contributions of both ligand and protein atoms to the total binding affinity. As expected from the analysis of the ligand-only model, protein contributions have a small magnitude compared to atoms in the ligand.

## Discussion

**Fig. 10:**
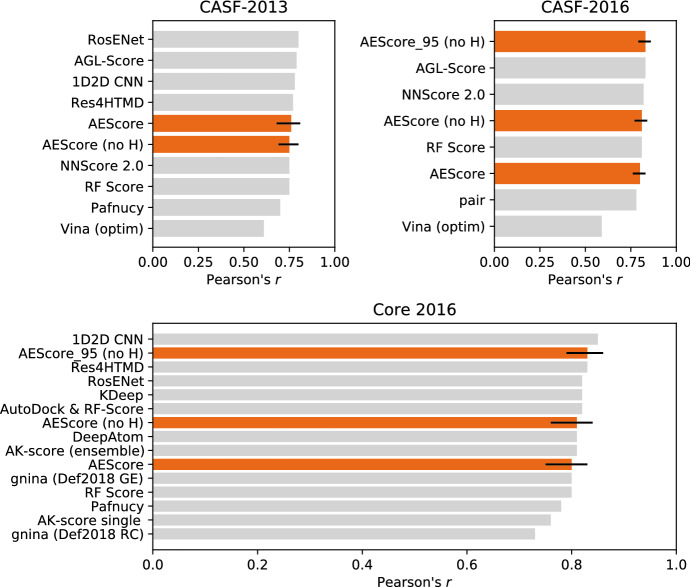
Performance of different machine learning and deep learning models for binding affinity prediction on the CASF-2013 and CASF-2016 benchmarks as well as for the Core 2016 set. Our results, shown in orange, include 90% confidence intervals. Numerical values for the Pearson’s correlation coefficient and the RMSE are reported in Table S3, together with references for all the different methods [4, 11, 20, 22–25, 52, 65, 66, 75–78]

Figure 10 compares the performance of our model—denoted AEScore—in terms of binding affinity prediction for the CASF-2013 and CASF-2016 benchmark datasets, with other state-of-the-art machine learning and deep learning models. The performance of the other methods is taken directly from the references reported. The same results are also reported in Additional file 1: Table S4, together with RMSEs and additional information about models and training datasets.

In the literature there is some confusion about the CASF benchmark and the PDBbind Core set, as indicated on the PDBbind website [79]. In Additional file 1: Table S4 we indicate which dataset has been used for testing. The CASF-2016 benchmark set contains 285 protein-ligand complexes while the PDBbind Core 2016 set contains 290 protein-ligand complexes (complexes 4MRW, 4MRZ, 4MSN, 5C1W, 4MSC, and 3CYX in PDBbind Core 2016 are not included in CASF-2016, while 1G2K is an additional complex not present in the Core set) [66].

Our results compare favourably with other state-of-the-art deep learning models based on feed-forward NNs or CNNs and machine learning scoring functions based on random forests on both the CASF-2016 and PDBbind Core 2016 test sets. However, a quantitative and statistically sound comparison with other methods is somewhat difficult because error bars and confidence intervals are often not reported.

One of the main advantages of the AEV-based approach is that it is translationally and rotationally invariant, thus removing an additional source of variability. This is not the case for scoring functions based on standard CNNs, where random translations and rotations of the input protein-ligand systems give different results, while our results would remain unchanged. Additional file 1: Figure S11 shows the variation in CNN-based predictions as a function of the angle of rotation for a particular complex. Data augmentation with random translations and rotations has proved to be essential to prevent overfitting and significantly improve training in CNN-based scoring functions [20, 21], but this is computationally expensive—another advantage of our approach.

In addition to being translationally and rotationally invariant, atomic environment vectors also require minimal information about the system. Only elements and atomic coordinates are needed by the model. Other methods often require additional information such as force-field parameters or specific atom types and are therefore limited by these parameters and underlying assumptions.

Compared to “classical” machine learning scoring functions, our method performs similarly to RF Score and other RF-based scoring functions [11, 77]. Despite recent advances in deep learning architectures, which consistently outperform “classical” machine learning algorithms in image recognition and natural language processing [15–19], RFs remain very competitive for binding affinity predictions. All top-performing machine learning and deep learning methods considered here achieve similar performance on the CASF benchmarks—as measured by Pearson’s correlation coefficient. This is likely due to the fact that errors in the experimental measurements of the binding affinity and the X-ray crystallographic coordinates of the protein-ligand complex set a theoretical upper limit on the maximal performance of scoring functions trained on such noisy data [38].

It is instructive to also compare the performance of our model with standard docking scoring functions. Here we used the AutoDock Vina [10] scoring function as implemented in smina [80] as a baseline. We see that our model outperforms the Vina scoring function for protein-ligand affinity predictions, as do other machine learning and deep learning approaches. This is expected since previous studies show that standard scoring functions do not perform very well in scoring and ranking power tests [38].

The removal of the systems in the CASF test set from the PDBbind Refined set used for training is common practice with machine learning and deep learning scoring functions and therefore ensures a fair comparison with other methods. However, it has been previously noted that the performance on the CASF set is not necessarily very indicative of a model’s ability to generalize, since this dataset samples the same regions of the chemical and target spaces as the PDBbind dataset [41, 52]. In order to better evaluate the ability of a model to generalize, we tested its performance when trained on a recently developed non-redundant training set [41]. We showed in Fig. 5 that the performance of AEScore deteriorates gradually when the similarity between the training set and the test set is reduced, in contrast with many other machine learning scoring functions that are severely inhibited by removing structurally redundant samples from the training set [41].

**Fig. 11:**
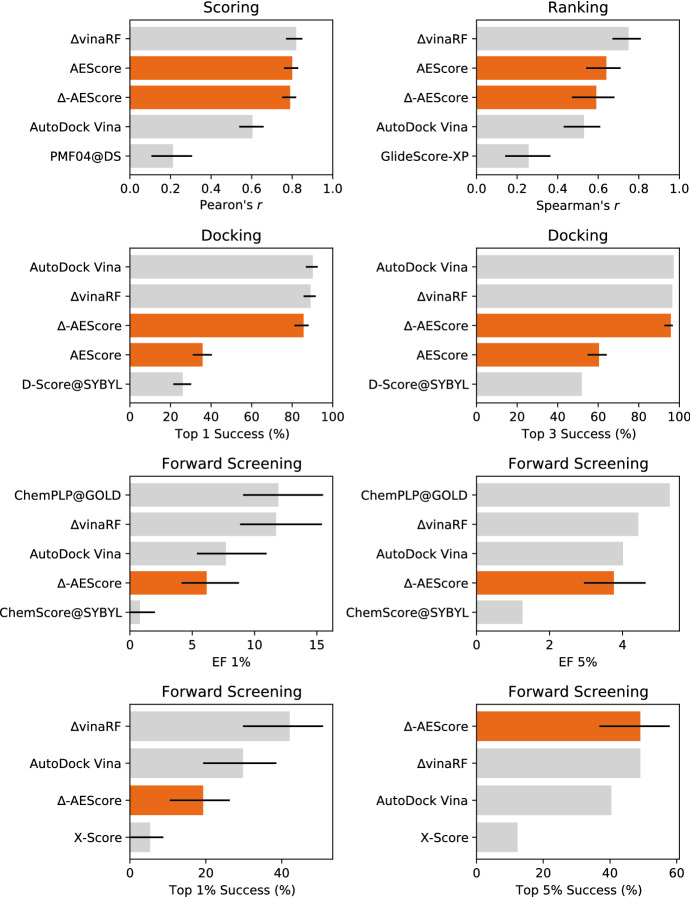
Performance of AEScore, $\Delta$-AEScore, $\Delta _\text {vina}\text {RF}$, and AutoDock Vina. The best- and worst-performing scoring functions on CASF-2016 (as reported by Su et al. [40]) are also added for comparison. The results include 90% confidence intervals (where they were available)

When we tested AEScore for docking power we obtained poor results. This is not surprising since the model was trained to predict binding affinities given the correct binding pose and it was not trained explicitly to distinguish low- from high-RMSD poses. However, we showed that by combining AEScore with the classical scoring function AutoDock Vina using a $\Delta$-learning approach improves the performance in terms of docking and screening while maintaining good scoring and ranking performance. As already demonstrated by $\Delta _\text {vina}\text {RF}$, this is a good approach for developing a scoring function that works well on all four tasks: scoring, ranking, docking, and screening. Usually, machine learning and deep learning scoring functions work very well for scoring but not as well for docking and virtual screening, while classical scoring functions have the opposite behaviour. Figure 11 collects most of the results of AEScore and $\Delta$-AEScore on the CASF-2016 benchmark, together with the results for $\Delta _\text {vina}\text {RF}$ and AutoDock Vina (our baseline) as reported by Su et al. [40]. We also added the best- and worst-performing scoring functions for each of the CASF-2016 benchmarks reported in Su et al. [40], whenever these scoring function were different from $\Delta _\text {vina}\text {RF}$ or AutoDock Vina. We see that both AEScore and $\Delta$-AEScore perform well in scoring and ranking power tests, but AEScore performance for docking is low. However, the $\Delta$-learning approach is able to recover a good docking power (similar to the AutoDock Vina baseline) while retaining a good performance in scoring and ranking. The performance of $\Delta$-AEScore in forward screening is rather poor as measured by EF 1% or top 1% success rate but greatly improves for EF 5% and the top 5% success rate.

Given the good performance of our ligand-only model—which was nonetheless consistently worse than that of the protein-ligand model—it is clear that the model is extracting a lot of information from the ligand. Finding strategies to force the model to rely more on protein information could further improve the model and make it more transferable. This is a known problem [68–70] and strategies to force the model to rely more on the protein structure are an active area of research [81].

The advantage of using an end-to-end differentiable model is that the gradient of the scoring function with respect to the input parameters can be readily obtained by backpropagation. Since the TorchANI AEVComputer is fully differentiable and its inputs are atomic coordinates [34], the gradient of the scoring function with respect to atomic coordinates can be computed. This can be used for visualization, which could help to understand the behaviour of the scoring function. In future iterations of the model, such gradients could be employed in the context of a local geometry optimization of the binding pose [74].

Finally, it is worth noting that we exploited the representation and architecture commonly used to develop NNP to predict a different endpoint, namely the protein-ligand binding affinity, and corrections to classical scoring functions. However, given the success of NNPs [28, 33] one could use them in a MM/PBSA- or MM/GBSA-style approach [82] to directly compute the free energy of binding on more physical grounds. In fact, approaches to combine NNP with molecular mechanics for drug discovery applications are already starting to appear [83–85].

## Conclusions

We demonstrated that AEVs are a promising representation of the protein-ligand binding site (and of the ligand alone, for ligand-based model) amenable to machine learning-based predictions of the protein-ligand binding affinity, and of corrections to classical scoring functions. This representation is rotationally and translationally invariant and, in contrast to CNN-based scoring functions, does not require data augmentation. The results reported here for AEScore show similar or better performance than other state-of-the-art machine learning and deep learning methods on the CASF-2013 and CASF-2016 benchmarks (as well as the Core 2016 set) in binding affinity prediction.

One of the major shortcomings of our model, however, is the over-reliance on ligand features as demonstrated by the good performance of the ligand-only model. This is a common problem with deep learning scoring functions [4, 52, 69, 70] and strategies to force the model to rely more on protein and ligand atoms involved in binding need to be developed [81].

Using training sets with decreasing similarity to the test set, first introduced by Boyles et al. [4] and later by Su et al. [41], we showed that our model is not completely hindered by the removal of systems with high similarity, but that AEScore’s performance deteriorates only gradually. This is in contrast with other machine learning and deep learning scoring functions, where a performance drop is observed as soon as a similarity threshold is introduced [4, 41]. This property could be useful in real drug discovery applications, where data on similar or related systems (such as a congeneric series of ligands) is acquired gradually.

In this work, we did not optimise the ANI parameters for radial and angular symmetry functions, and we did not explore the full flexibility of the angular symmetry functions. Bayesian optimisation of ACSFs’ hyperparameter space could lead to further improvements of the scoring function.

We also showed that the AEScore model presented here can be exploited in tandem with standard docking scoring functions using a $\Delta$-learning approach, in order to improve the performance in docking and virtual screening (in which AEScore does not perform well, since it has not been explicitly trained for such task). $\Delta$-AEScore outperforms the $\Delta _\text {vina}\text {RF}$ scoring function by a good margin on the CASF-2013 test set and performs similarly on the CASF-2016 test set (notwithstanding the training/test set overlap in $\Delta _\text {vina}\text {RF}$ reported performance). $\Delta$-learning has the advantage of partially retaining the good docking and screening power of standard scoring functions while improving affinity predictions using machine-learning corrections, allowing the development of a scoring function that works reasonably well on all four tasks of early-stage structure-based drug discovery applications.

## Supplementary Information

**Additional file 1.** Supplementary information.

## Data Availability

The code for training and inference is available on GitHub (https://github.com/bigginlab/aescore) while data and scripts for the numerical experiments are available on Zenodo (https://doi.org/10.5281/zenodo.4155365).

## References

[1] Aldeghi M, Heifetz A, Bodkin MJ, Knapp S, Biggin PC (2016). Accurate calculation of the absolute free energy of binding for drug molecules. Chem Sci.

[2] Aldeghi M, Bluck JP, Biggin PC, Gore M, Jagtap UB (2018). Absolute alchemical free energy calculations for ligand binding: a beginner’s guide. Methods in molecular biology.

[3] Kitchen DB, Decornez H, Furr JR, Bajorath J (2004). Docking and scoring in virtual screening for drug discovery: methods and applications. Nat Rev Drug Discov.

[4] Boyles F, Deane CM, Morris GM (2019). Learning from the ligand: using ligand-based features to improve binding affinity prediction. Bioinformatics.

[5] Liu J, Wang R (2015). Classification of current scoring functions. J Chem Inf Model.

[6] Jones G, Willett P, Glen RC, Leach AR, Taylor R (1997) Development and validation of a genetic algorithm for flexible docking 1 1Edited by F. E. Cohen. J Mol Biol 267(3):727–748. 10.1006/jmbi.1996.0897 10.1006/jmbi.1996.0897 9126849

[7] Halgren TA, Murphy RB, Friesner RA, Beard HS, Frye LL, Pollard WT, Banks JL (2004). Glide: a new approach for rapid, accurate docking and scoring. 2. Enrichment factors in database screening. J Med Chem.

[8] Morris GM, Huey R, Lindstrom W, Sanner MF, Belew RK, Goodsell DS, Olson AJ (2009). AutoDock4 and AutoDockTools4: automated docking with selective receptor flexibility. J Comput Chem.

[9] Ravindranath PA, Forli S, Goodsell DS, Olson AJ, Sanner MF (2015). AutoDockFR: advances in protein-ligand docking with explicitly specified binding site flexibility. PLoS Comput Biol.

[10] Trott O, Olson AJ (2009). AutoDock vina: improving the speed and accuracy of docking with a new scoring function, efficient optimization, and multithreading. J Comput Chem.

[11] Ballester PJ, Mitchell JBO (2010). A machine learning approach to predicting protein-ligand binding affinity with applications to molecular docking. Bioinformatics.

[12] Ain QU, Aleksandrova A, Roessler FD, Ballester PJ (2015). Machine-learning scoring functions to improve structure-based binding affinity prediction and virtual screening. WIREs Comput Mol Sci.

[13] Cherkasov A, Muratov EN, Fourches D, Varnek A, Baskin II, Cronin M, Dearden J, Gramatica P, Martin YC, Todeschini R, Consonni V, Kuz’min VE, Cramer R, Benigni R, Yang C, Rathman J, Terfloth L, Gasteiger J, Richard A, Tropsha A (2014). QSAR modeling: Where have you been? Where are you going to?. J Med Chem.

[14] Muratov EN, Bajorath J, Sheridan RP, Tetko IV, Filimonov D, Poroikov V, Oprea TI, Baskin II, Varnek A, Roitberg A, Isayev O, Curtalolo S, Fourches D, Cohen Y, Aspuru-Guzik A, Winkler DA, Agrafiotis D, Cherkasov A, Tropsha A (2020). QSAR without borders. Chem Soc Rev.

[15] Krizhevsky A, Sutskever I, Hinton GE (2012) ImageNet classification with deep convolutional neural networks. In: Pereira F, Burges CJC, Bottou L, Weinberger KQ eds. Advances in neural information processing systems. Curran Associates, Inc., Red Hook, vol 25, pp 1097–1105.

[16] Krizhevsky A, Sutskever I, Hinton GE (2017). ImageNet classification with deep convolutional neural networks. Commun ACM.

[17] Graves A, Mohamed A-R, Hinton G (2013) Speech recognition with deep recurrent neural networks. arXiv:1303.5778 [cs]

[18] Hinton G, Deng L, Yu D, Dahl G, Mohamed A-R, Jaitly N, Senior A, Vanhoucke V, Nguyen P, Sainath T, Kingsbury B (2012). Deep neural networks for acoustic modeling in speech recognition: the shared views of four research groups. IEEE Signal Process Mag.

[19] LeCun Y, Bengio Y, Hinton G (2015). Deep learning. Nature.

[20] Ragoza M, Hochuli J, Idrobo E, Sunseri J, Koes DR (2017). Protein-ligand scoring with convolutional neural networks. J Chem Inf Model.

[21] McNutt AT, Francoeur P, Aggarwal R, Masuda T, Meli R, Ragoza M, Sunseri J, Koes DR (2021). Gnina 1.0: molecular docking with deep learning. J Cheminform.

[22] Stepniewska-Dziubinska MM, Zielenkiewicz P, Siedlecki P (2018). Development and evaluation of a deep learning model for protein-ligand binding affinity prediction. Bioinformatics.

[23] Jiménez J, Škalič M, Martínez-Rosell G, De Fabritiis G (2018). KDEEP: protein-ligand absolute binding affinity prediction via 3D-convolutional neural networks. J Chem Inf Model.

[24] Cang Z, Mu L, Wei G-W (2018). Representability of algebraic topology for biomolecules in machine learning based scoring and virtual screening. PLoS Comput Biol.

[25] Hassan-Harrirou H, Zhang C, Lemmin T (2020). RosENet: improving binding affinity prediction by leveraging molecular mechanics energies with an ensemble of 3D convolutional neural networks. J Chem Inf Model.

[26] Feinberg EN, Sur D, Wu Z, Husic BE, Mai H, Li Y, Sun S, Yang J, Ramsundar B, Pande VS (2018). PotentialNet for molecular property prediction. ACS Cent Sci.

[27] Behler J, Parrinello M (2007). Generalized neural-network representation of high-dimensional potential-energy surfaces. Phys Rev Lett.

[28] Smith JS, Isayev O, Roitberg AE (2017). ANI-1: an extensible neural network potential with DFT accuracy at force field computational cost. Chem Sci.

[29] Bartók AP, Payne MC, Kondor R, Csányi G (2010). Gaussian approximation potentials: the accuracy of quantum mechanics, without the electrons. Phys Rev Lett.

[30] Bartók AP, Kondor R, Csányi G (2013). On representing chemical environments. Phys. Rev. B.

[31] Bartók AP, De S, Poelking C, Bernstein N, Kermode JR, Csányi G, Ceriotti M (2017). Machine learning unifies the modeling of materials and molecules. Sci Adv.

[32] Behler J (2011). Neural network potential-energy surfaces in chemistry: a tool for large-scale simulations. Phys Chem Chem Phys.

[33] Smith JS, Nebgen B, Lubbers N, Isayev O, Roitberg AE (2018). Less is more: Sampling chemical space with active learning. J. Chem. Phys..

[34] Gao X, Ramezanghorbani F, Isayev O, Smith JS, Roitberg AE (2020). TorchANI: a free and open source PyTorch-based deep learning implementation of the ANI neural network potentials. J Chem Inf Model.

[35] Paszke A, Gross S, Massa F, Lerer A, Bradbury J, Chanan G, Killeen T, Lin Z, Gimelshein N, Antiga L, Desmaison A, Kopf A, Yang E, DeVito Z, Raison M, Tejani A, Chilamkurthy S, Steiner B, Fang L, Bai J, Chintala S (2019) PyTorch: An imperative style high-performance deep learning library, In: Wallach H, Larochelle H, Beygelzimer A, d’ Alché-Buc F, Fox E, Garnett R, (eds) Advances in neural information processing systems. Curran Associates, Inc., vol 32, pp. 8024–8035. http://papers.neurips.cc/paper/9015-ytorch-an-imperative-style-high-performance-deep-learning-library.pdf

[36] Smith JS, Nebgen BT, Zubatyuk R, Lubbers N, Devereux C, Barros K, Tretiak S, Isayev O, Roitberg AE (2019). Approaching coupled cluster accuracy with a general-purpose neural network potential through transfer learning. Nat Commun.

[37] Li Y, Liu Z, Li J, Han L, Liu J, Zhao Z, Wang R (2014). Comparative assessment of scoring functions on an updated benchmark: 1. compilation of the test set. J Chem Inf Model.

[38] Liu Z, Su M, Han L, Liu J, Yang Q, Li Y, Wang R (2017). Forging the basis for developing protein-ligand interaction scoring functions. Acc Chem Res.

[39] Li Y, Han L, Liu Z, Wang R (2014). Comparative assessment of scoring functions on an updated benchmark: 2. evaluation methods and general results. J. Chem. Inf. Model..

[40] Su M, Yang Q, Du Y, Feng G, Liu Z, Li Y, Wang R (2018). Comparative assessment of scoring functions: the CASF-2016 update. J Chem Inf Model.

[41] Su M, Feng G, Liu Z, Li Y, Wang R (2020). Tapping on the black box: how is the scoring power of a machine-learning scoring function dependent on the training set?. J Chem Inf Model.

[42] O’Boyle NM, Banck M, James CA, Morley C, Vandermeersch T, Hutchison GR (2011). Open babel: an open chemical toolbox. JCheminform.

[43] Michaud-Agrawal N, Denning EJ, Woolf TB, Beckstein O (2011). MDAnalysis: a toolkit for the analysis of molecular dynamics simulations. J Comput Chem.

[44] Gowers RJ, Linke M, Barnoud J, Reddy TJE, Melo MN, Seyler SL, Domański J, Dotson, D.L., Buchoux, S., Kenney, I.M., Beckstein, O (2016) MDAnalysis: A Python Package for the Rapid Analysis of Molecular Dynamics Simulations. In: Sebastian Benthall, Scott Rostrup (eds.) Proceedings of the 15th Python in science conference, pp. 98–105 . 10.25080/Majora-629e541a-00e

[45] O’Boyle NM, Morley C, Hutchison GR (2008) Pybel: A python wrapper for the OpenBabel cheminformatics toolkit. Chem. Cent. J. 2(1):5. 10.1186/1752-153x-2-5 10.1186/1752-153X-2-5 PMC2270842 18328109

[46] Banci L (2003). Molecular dynamics simulations of metalloproteins. Curr Opin Chem Biol.

[47] Çınaroğlu SS, Timuçin E (2019). Comparative assessment of seven docking programs on a nonredundant metalloprotein subset of the PDBbind refined. J Chem Inf Model.

[48] Ramakrishnan R, Dral PO, Rupp M, von Lilienfeld OA (2015). Big data meets quantum chemistry approximations: the $$\delta$$-machine learning approach. J Chem Theory Comput.

[49] Wang C, Zhang Y (2016). Improving scoring-docking-screening powers of protein-ligand scoring functions using random forest. J Comput Chem.

[50] Lu J, Hou X, Wang C, Zhang Y (2019). Incorporating explicit water molecules and ligand conformation stability in machine-learning scoring functions. J Chem Inf Model.

[51] Ericksen SS, Wu H, Zhang H, Michael LA, Newton MA, Hoffmann FM, Wildman SA (2017). Machine learning consensus scoring improves performance across targets in structure-based virtual screening. J Chem Inf Model.

[52] Francoeur PG, Masuda T, Sunseri J, Jia A, Iovanisci RB, Snyder I, Koes DR (2020). Three-dimensional convolutional neural networks and a cross-docked data set for structure-based drug design. J Chem Inf Model.

[53] Van Rossum G, Drake FL (2009). Python 3 reference manual.

[54] O’Boyle NM, Banck M, James CA, Morley C, Vandermeersch T, Hutchison GR (2011). Open babel: an open chemical toolbox. J Cheminform.

[55] Harris CR, Millman KJ, van der Walt SJ, Gommers R, Virtanen P, Cournapeau D, Wieser E, Taylor J, Berg S, Smith NJ, Kern R, Picus M, Hoyer S, van Kerkwijk MH, Brett M, Haldane A, del Río JF, Wiebe M, Peterson P, Gérard-Marchant P, Sheppard K, Reddy T, Weckesser W, Abbasi H, Gohlke C, Oliphant TE (2020). Array programming with NumPy. Nature.

[56] Virtanen P, Gommers R, Oliphant TE, Haberland M, Reddy T, Cournapeau D, Burovski E, Peterson P, Weckesser W, Bright J, van der Walt SJ, Brett M, Wilson J, Millman KJ, Mayorov N, Nelson ARJ, Jones E, Kern R, Larson E, Carey CJ, Polat İ, Feng Y, Moore EW, VanderPlas J, Laxalde D, Perktold J, Cimrman R, Henriksen I, Quintero EA, Harris CR, Archibald AM, Ribeiro AH, Pedregosa F, van Mulbregt P, Vijaykumar A, Bardelli AP, Rothberg A, Hilboll A, Kloeckner A, Scopatz A, Lee A, Rokem A, Woods CN, Fulton C, Masson C, Häggström C, Fitzgerald C, Nicholson DA, Hagen DR, Pasechnik DV, Olivetti E, Martin E, Wieser E, Silva F, Lenders F, Wilhelm F, Young G, Price GA, Ingold G-L, Allen GE, Lee GR, Audren H, Probst I, Dietrich JP, Silterra J, Webber JT, Slavič J, Nothman J, Buchner J, Kulick J, Schönberger JL, de Miranda Cardoso J, Reimer J, Harrington J, Rodríguez JLC, Nunez-Iglesias J, Kuczynski J, Tritz K, Thoma M, Newville M, Kümmerer M, Bolingbroke M, Tartre M, Pak M, Smith NJ, Nowaczyk N, Shebanov N, Pavlyk O, Brodtkorb PA, Lee P, McGibbon RT, Feldbauer R, Lewis S, Tygier S, Sievert S, Vigna S, Peterson S, More S, Pudlik T, Oshima T, Pingel TJ, Robitaille TP, Spura T, Jones TR, Cera T, Leslie T, Zito T, Krauss T, Upadhyay U, Halchenko YO, Vázquez-Baeza Y, Contributors S (2020). SciPy 1.0: Fundamental algorithms for scientific computing in python. Nat Methods.

[57] McKinney W (2010) Data Structures for Statistical Computing in Python. In: van der Walt S, Millman J (eds.) Proceedings of the 9th Python in science conference, pp. 56–61. 10.25080/Majora-92bf1922-00a

[58] Hunter JD (2007). Matplotlib: a 2D graphics environment. Comput Sci Eng.

[59] Waskom M (2020). The seaborn development team: Mwaskom/seaborn. Zenodo.

[60] Varoquaux G, Buitinck L, Louppe G, Grisel O, Pedregosa F, Mueller A (2015) Scikit-learn. GetMobile: Mobile Comp and Comm 19(1):29–33. 10.1145/2786984.2786995

[61] Krekel H, Oliveira B, Pfannschmidt R, Bruynooghe F, Laugher B, Bruhin F (2004) pytest 6.0. https://github.com/pytest-dev/pytest

[62] Kingma DP, Ba J (2014) ADAM: a method for stochastic optimization. arXiv:1412.6980

[63] Ross GA, Morris GM, Biggin PC (2013). One size does not fit all: the limits of structure-based models in drug discovery. J Chem Theory Comput.

[64] Pearlman DA, Charifson PS (2001). Are free energy calculations useful in practice? a comparison with rapid scoring functions for comparison with rapid scoring functions the p38 MAP kinase protein system$$\dagger$$. J Med Chem.

[65] Kwon Y, Shin W-H, Ko J, Lee J (2020). AK-Score: accurate protein-ligand binding affinity prediction using the ensemble of 3D-convolutional neural network. Int J Mol Sci.

[66] Nguyen DD, Wei G-W (2019). AGL-score: algebraic graph learning score for protein-ligand binding scoring, ranking, docking, and screening. J Chem Inf Model.

[67] Cheng T, Li X, Li Y, Liu Z, Wang R (2009). Comparative assessment of scoring functions on a diverse test set. J Chem Inf Model.

[68] Sieg J, Flachsenberg F, Rarey M (2019). In need of bias control: evaluating chemical data for machine learning in structure-based virtual screening. J Chem Inf Model.

[69] Wallach I, Heifets A (2018). Most ligand-based classification benchmarks reward memorization rather than generalization. J Chem Inf Model.

[70] Chen L, Cruz A, Ramsey S, Dickson CJ, Duca JS, Hornak V, Koes DR, Kurtzman T (2019). Hidden bias in the DUD-e dataset leads to misleading performance of deep learning in structure-based virtual screening. PLoS One.

[71] Musil F, Grisafi A, Bartók AP, Ortner C, Csányi G, Ceriotti M (2021) Physics-inspired structural representations for molecules and materials . arXiv:2101.04673 10.1021/acs.chemrev.1c00021 34310133

[72] McCorkindale W, Poelking C, Lee AA (2020) Investigating 3D Atomic Environments for Enhanced QSAR. arXiv:2010.12857

[73] Hochuli J, Helbling A, Skaist T, Ragoza M, Koes DR (2018). Visualizing convolutional neural network protein-ligand scoring. J Mol Graph Model.

[74] Ragoza M, Turner L, Koes DR (2017) Ligand pose optimization with atomic grid-based convolutional neural networks . arXiv:1710.07400

[75] Durrant JD, McCammon JA (2011). NNScore 2.0: a neural-network Receptor-Ligand scoring function. J Chem Inf Model.

[76] Zhu F, Zhang X, Allen JE, Jones D, Lightstone FC (2020). Binding affinity prediction by pairwise function based on neural network. J Chem Inf Model.

[77] Afifi K, Al-Sadek AF (2018). Improving classical scoring functions using random forest: the non-additivity of free energy terms’ contributions in binding. Chem Biol Drug Des.

[78] Li Y, Rezaei MA, Li C, Li X, Wu D (2019) DeepAtom: a Framework for protein-ligand binding affinity prediction . arXiv:1912.00318

[79] PDBbind-CN Database. http://pdbbind-cn.org/

[80] Koes DR, Baumgartner MP, Camacho CJ (2013). Lessons learned in empirical scoring with smina from the CSAR 2011 benchmarking exercise. J Chem Inf Model.

[81] Scantlebury J, Brown N, Von Delft F, Deane CM (2020). Data set augmentation allows deep learning-based virtual screening to better generalize to unseen target classes and highlight important binding interactions. J Chem Inf Model.

[82] Genheden S, Ryde U (2015). The MM/PBSA and MM/GBSA methods to estimate ligand-binding affinities. Expert Opin Drug Discov.

[83] Cole DJ, Mones L, Csányi G (2020). A machine learning based intramolecular potential for a flexible organic molecule. Faraday Discuss.

[84] Lahey S-LJ, Rowley CN (2020). Simulating protein-ligand binding with neural network potentials. Chem Sci.

[85] Rufa DA, Bruce Macdonald HE, Fass J, Wieder M, Grinaway PB, Roitberg AE, Isayev O, Chodera JD (2020). Towards chemical accuracy for alchemical free energy calculations with hybrid physics-based machine learning/molecular mechanics potentials. bioRxiv.

