# Supplementary material for: Learning protein-ligand binding affinity with atomic environment vectors
Source: J Cheminform. 2021 Aug 14;13:59. doi: 10.1186/s13321-021-00536-w (PMC8364054; doi:10.1186/s13321-021-00536-w)
Supplement: Supplementary file 1 — Additional file 1. Supplementary information. [file 13321_2021_536_MOESM1_ESM.pdf]

Supplementary Information: Learning Protein-Ligand Binding Affinity with Atomic Environment Vectors

Rocco Meli,[†] Andrew Anighoro,[‡] Mike J. Bodkin,[‡] Garrett M. Morris,[*,¶] and Philip C. Biggin[*,†]

†*Department of Biochemistry, University of Oxford, South Parks Rd., Oxford, OX1 3QU, UK.*

‡*Evotec (UK) Ltd., 114 Innovation Drive, Milton Park, Abingdon, Oxfordshire OX14 4RZ, UK*

¶*Department of Statistics, University of Oxford, 24-29 St. Giles', Oxford, OX1 3LB, UK.*

E-mail: garrett.morris@dtc.ox.ac.uk; philip.biggin@bioch.ox.ac.uk

Example of Atomic Environment Vector Computation

Water

As a simple example of how atom-centred symmetry functions (ACFSs) are used to construct atomic environment vectors (AEVs), let us consider a simple water molecule with the fictitious coordinates of Table 1

Table 1: Fictitious coordinates for a water molecule.

	index	x	y	z
H	1	1	0	0
H	2	0	1	0
O	3	0	0	0

If this is the only system we want to describe, we have only two elements ($N_\mathrm{e} = 2$) and we need to compute three AEVs, one for each atom.

Radial symmetry functions are parametrised by η_R and R_s; for simplicity we only consider here $\eta_R = 1$ and $R_s = \{0, 1\}$. Angular symmetry functions are parametrised by η_A, R_s, θ_s and ζ; for simplicity we only consider here $\eta_A = 1$, $R_s = 0$, $\theta_s = 0$ and $\zeta = 1$.

The atomic environment vector for the oxygen atom (for $N_e = 2$) has the following form:

$$\mathbf{G}_3^O = [G^R_{3;H,R_s=0}, G^R_{3;H,R_s=1}, G^R_{3,O,R_s=0}, G^R_{3,O,R_s=1}; G^A_{3;H,H}, G^A_{3;H,O}, G^A_{3;O,O}]$$

Since there are no other oxygen atoms in the system, we have $G^R_{3,O,R_s=0} = 0$, $G^A_{3,H,O} = 0$, and $G^A_{3,O,O} = 0$ since such ACSFs depend on one or two neighbouring oxygen atoms within the cutoff distance R_c (which we consider here large enough to include all atoms of the system). The atomic environment vector for the oxygen atom therefore reduces to

$$\mathbf{G}_3^O = [G^R_{3;H,R_s=0}, G^R_{3;H,R_s=1}, 0, 0; G^A_{3;H,H}, 0, 0].$$

Explicitly, the non-zero ACSFs composing the AEV for the oxygen atom are

$$G^R_{3;H,R_s=0} = \sum_{\substack{j\neq 3\\ j\in H}} e^{-R^2_{3,j}} f_c(R_{3,j}) = e^{-R^2_{3,1}} f_c(R_{3,1}) + e^{-R^2_{3,2}} f_c(R_{3,2})$$

$$G^R_{3;H,R_s=1} = \sum_{\substack{j\neq 3\\ j\in H}} e^{-(R_{3,j}-1)^2} f_c(R_{3,j}) = e^{-(R_{3,1}-1)^2} f_c(R_{3,1}) + e^{-(R_{3,2}-1)^2} f_c(R_{3,2})$$

$$\begin{aligned} G^A_{3;H,H} &= \sum_{\substack{j,k\neq 3\\ j\in H,k\in H}} [1+\cos(\theta_{3,j,k})] e^{-\left(\frac{R_{3,j}+R_{3,k}}{2}\right)^2} f_c(R_{3,j}) f_c(R_{3,k}) \\ &= [1+\cos(\theta_{3,1,2})] e^{-\left(\frac{R_{3,1}+R_{3,2}}{2}\right)^2}] f_c(R_{3,1}) f_c(R_{3,2}) \end{aligned}$$

If we consider R_c to be large enough so that $f_c(R) \approx 1$ and we use the geometry defined in Table 1, we can perform an explicit calculation for the particular configuration considered here (where $R_{3,1} = R_{3,2} = 1$ and $\theta_{3,1,2} = \pi/2$):

$$\mathbf{G}^O_3 = [2e^{-1}, 2, 0, 0; e^{-1}, 0, 0]$$

The AEV for oxygen encodes its atomic environment and it is, by construction, rotationally and translationally invariant.

The same procedure can be repeated for every atom of the system, so that all atoms are described by their own AEV, so that we can describe the whole system with a matrix of AEVs of dimension $N_{\text{atoms}} \times N_{\text{AEVs}}$ (where N_{AEVs} depends on the number of elements n_e as well as the number of different values for the parameters R_s, η_R, ...).

Fig. 1 shows the atomic environment vectors for water computed with TorchANI.[1] Discrepancies with the analytical calculation above come from the fact that the radial part is multiplied by a factor of 1/4 in the TorchANI implementation (see TorchANI code).

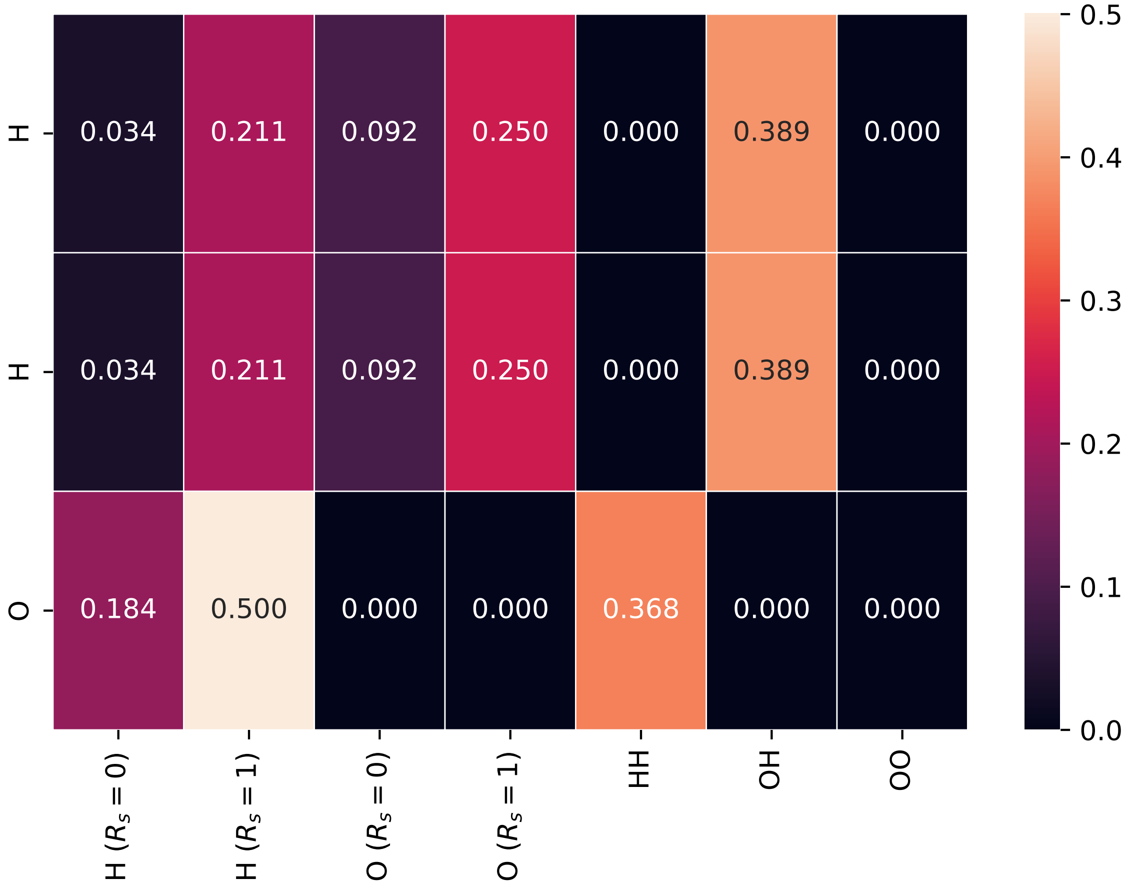

Figure 1: AEVs for the atoms in the water molecule defined in Tab 1. The two hydrogen atoms have the same AEVs because of symmetry.

Ammonia

Let us consider another simple example: ammonia. Again, we only have two elements ($N_e = 2$) and we need to compute four AEVs, one for each atom. If we consider the same parametrisation for radial and angular symmetry functions described above, we have the following atomic environment vector for nitrogen:

$$\mathbf{G}^N = [G^R_{N;H,R_s=0}, G^R_{N;H,R_s=1}, G^R_{N;N,R_s=0}, G^R_{N;N,R_s=1}; G^A_{N;H,H}, G^A_{N;H,N}, G^A_{N;N,N}]$$

since there are no other nitrogen atoms in the system, the AEV for the only nitrogen atom reduces to

$$\mathbf{G}^N = [G^R_{N;H,R_s=0}, G^R_{N;H,R_s=1}, 0, 0; G^A_{N;H,H}, 0, 0]$$

Explicitly, denoting d_{NH} the nitrogen-hydrogen distance, we have

$$G^R_{N;H,R_s=0} = \sum_{\substack{j \neq N \\ j \in H}} e^{-d_{NH}^2} f_c(d_{NH}) = 3e^{-d_{NH}^2} f_c(d_{NH})$$

$$G^R_{N;H,R_s=1} = \sum_{\substack{j \neq N \\ j \in H}} e^{-(d_{NH}-1)^2} f_c(d_{NH}) = 3e^{-(d_{NH}-1)^2} f_c(d_{NH})$$

$$\begin{aligned} G^A_{N;H,H} &= \sum_{\substack{j,k \neq N \\ j \in H, k \in H}} [1 + \cos(\theta_{N;HH})] e^{-\left(\frac{d_{NH}+d_{NH}}{2}\right)^2} f_c(d_{NH}) f_c(d_{NH}) \\ &= 3[1 + \cos(\theta_{N;HH})] e^{-(d_{NH})^2} f_c^2(d_{NH}) \end{aligned}$$

Using $d_{NH} = 1$ and $\theta_{N;HH} = 109.5$ we have the following atomic environment vector for nitrogen

$$\mathbf{G}^N = \left[3e^{-1}, 3, 0, 0; 3\left(1 + \cos(109.5)\right) e^{-1}, 0, 0\right].$$

Fig. 2 shows the atomic environment vectors for ammonia computed with TorchANI.[1]

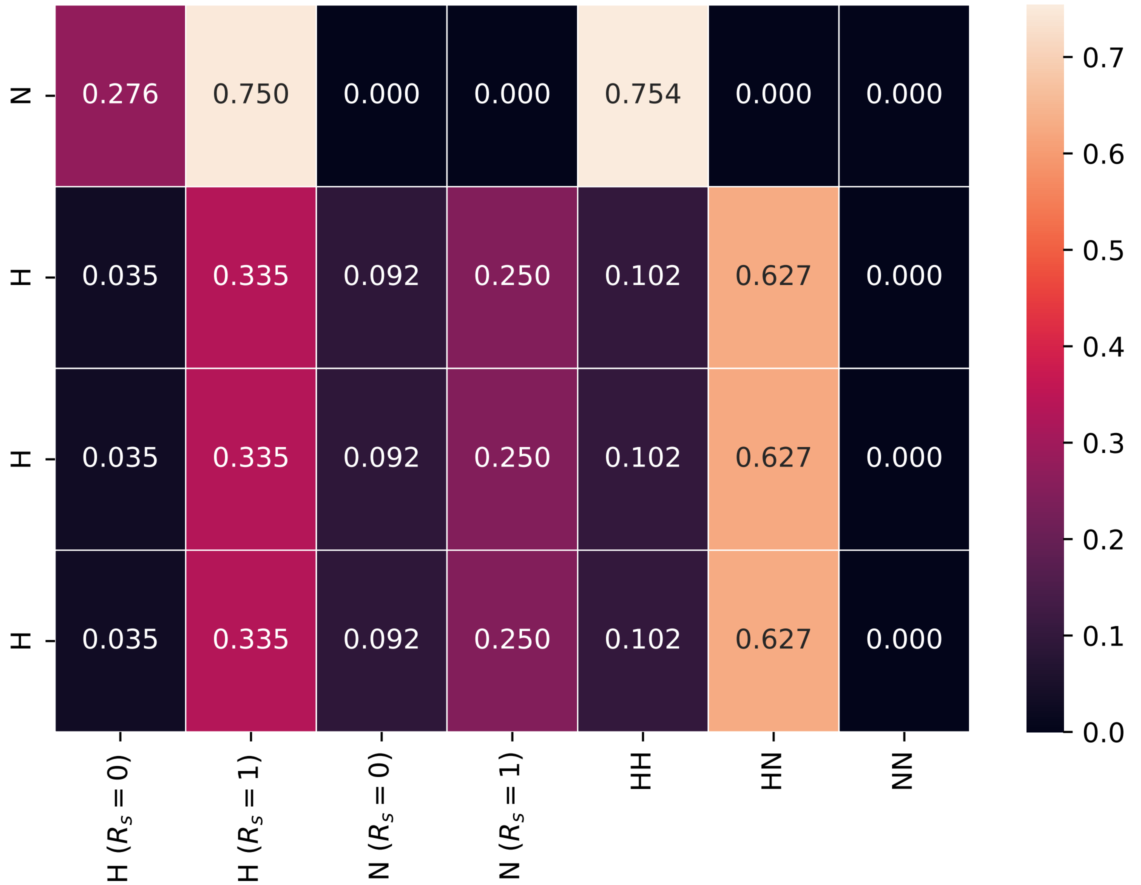

Figure 2: AEVs for the atoms in the ammonia molecule defined in Tab 1. The three hydrogen atoms have the same AEVs because of symmetry.

Discrepancies with the analytical calculation above come from the fact that the radial part is multiplied by a factor of 1/4 in the TorchANI implementation (see TorchANI code) and that the angle between two vectors is computed as $\theta = \arccos(0.95 * c)$ where $c = \frac{\vec{v}_1 \cdot \vec{v}_2}{|\vec{v}_1||\vec{v}_2|}$ instead of $\theta = \arccos(c)$ (see TorchANI code).

Gradients

The model, consisting of the AEVComputer and a collection of NNs, can be described as a function of the atomic coordinates $f(\vec{R})$, returning the binding affinity a:

$$a = f(\vec{R})$$

The loss function (MSE loss) is defined as the mean square difference between predicted and experimental affinities. For a single prediction a and the corresponding experimental affinity A, the loss function is:

$$\mathcal{L}(a, A) = (a - A)^2$$

Given that $a = f(\vec{R})$, the gradient of the loss function with respect to the atomic coordinates can be computed using the chain rule

$$\nabla_{\vec{R}} \mathcal{L}(a, A) = \nabla_{\vec{R}} (a(\vec{R}) - A)^2 = 2(a - A) \nabla_{\vec{R}} a = 2(a - A) \nabla_{\vec{R}} f(\vec{r})$$

The negative gradient of the loss function indicates the directions where the atoms can be moved to minimise the loss function, i.e. bring the predicted binding affinity a closer to the expected binding affinity A.

In some cases, such as classification of actives and decoys, it is clear what the desired outcome of the model is (i.e. an active molecule) and therefore the loss can be computed with respect to the desired output. In other cases, such as the prediction of the binding affinity, the desired outcome A (experimental binding affinity) is usually not known, and therefore it is not possible to compute the loss and its gradient. However, it remains possible to compute the gradient of the output with respect to the atomic coordinates.

The gradient of the predicted binding affinity a can be computed by differentiating f

with respect to the atomic coordinates $\vec{R}$:

$$\nabla_{\vec{R}} a = \nabla_{\vec{R}} f(\vec{R}).$$

This gradient can be computed with back-propagation and only requires a forward pass within the network. The positive gradient of the input indicates the directions where the atoms can be moved in order to increase (maximise) the predicted binding affinity.

For convenience of visualization, it is possible to show the (magnitude) of the gradient on each atom as color-coded.

Table 2: Model performance—with consensus scoring—on the validation set for different values of d, all else being fixed to optimal values (256-128-64-1 feed-forward atomic NNs, batch size of 64 and dropout probability of 25%). The approximate training time per epoch is also reported. Training is performed on an NVIDIA GeForce GTX 1080 Ti GPU.

Distance (Å)	RMSE	Pearson's r	Time (s/epoch)
0.0	1.52	0.72	1.6
2.5	1.51	0.74	3.3
3.0	1.42	0.76	5.0
3.5	1.37	0.78	5.7
4.0	1.35	0.78	6.3

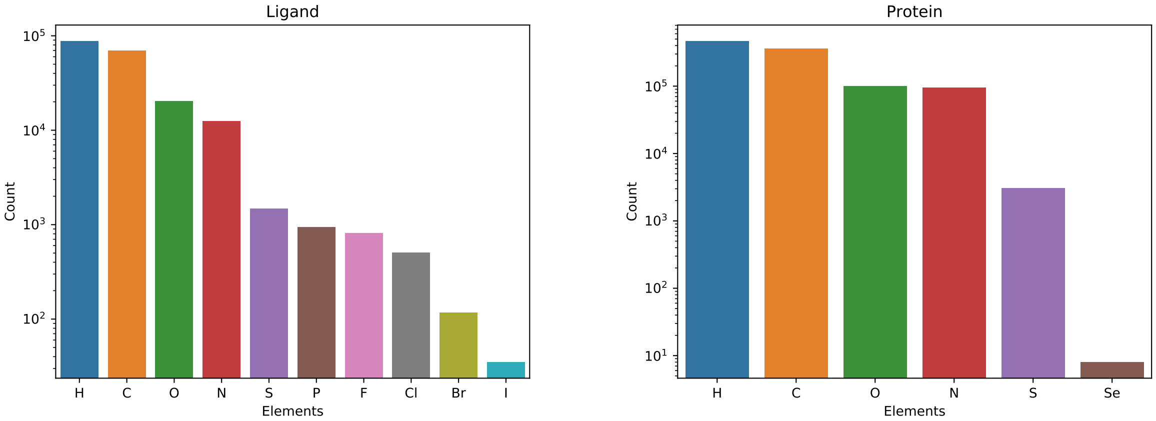

(a) Ligand (b) Protein

Figure 3: Number of atoms—within $d = 3.5$ Å from the ligand—for each element in the PDBbind 2016 refined set[2] when only protein residues are considered.

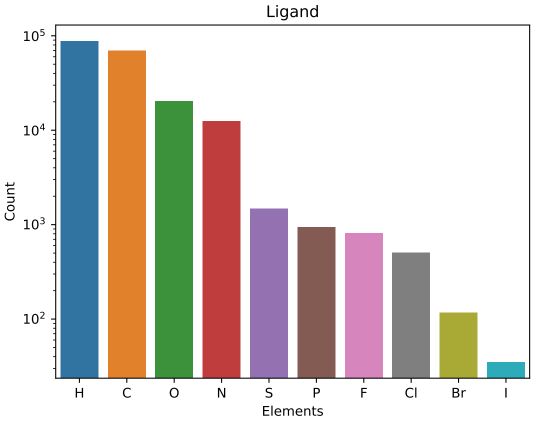

(a) Ligand

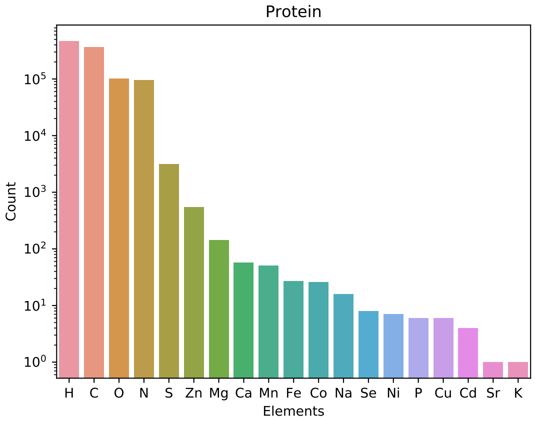

(b) Protein

Figure 4: Number of atoms—within d = 3.5 Å from the ligand—for each element in the PDBbind 2016 refined set.[2] The following PDB IDs correspond to selenoproteins: 1uj6, 1nu3, 3gpo, 2wqp, 3m89, 3hx3, 2qry.

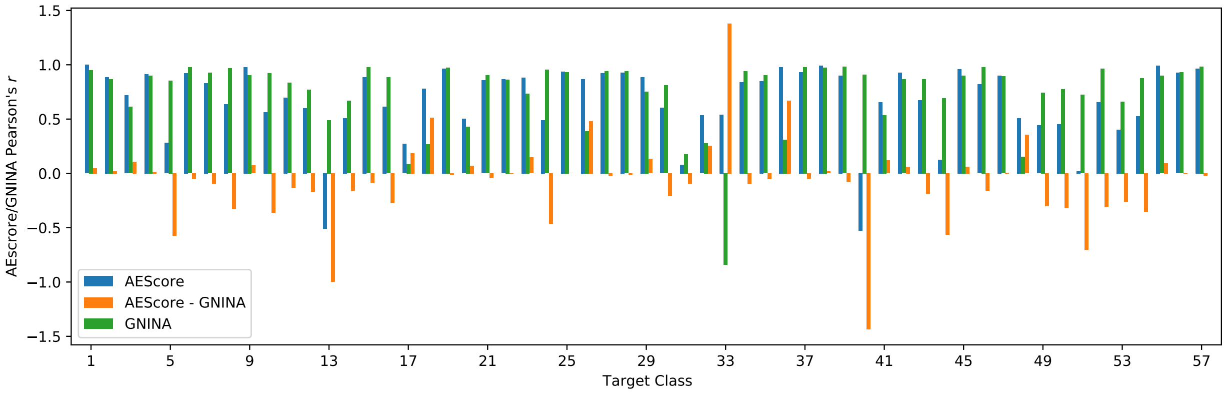

Figure 5: Comparison of per-class Pearson correlation coefficient between AEScore and GNINA.[3,4] The difference in correlation coefficient between the two methods (AEScore − GNINA) is also reported.

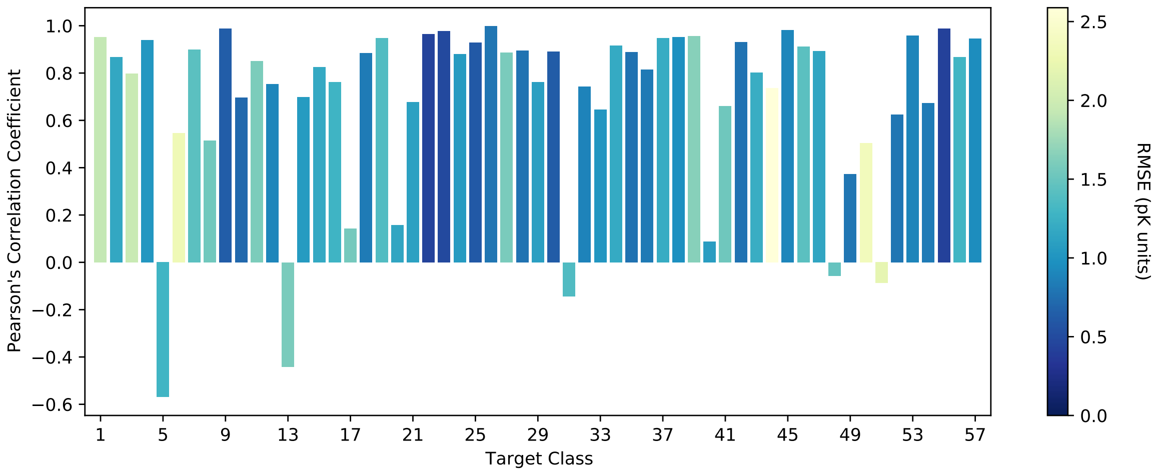

Figure 6: Per-class Pearson correlation coefficient, with each bar color-coded by the corresponding RMSE in pK units, for the 57 classes of the CASF-2016 dataset. The model is trained on systems without hydrogen atoms.

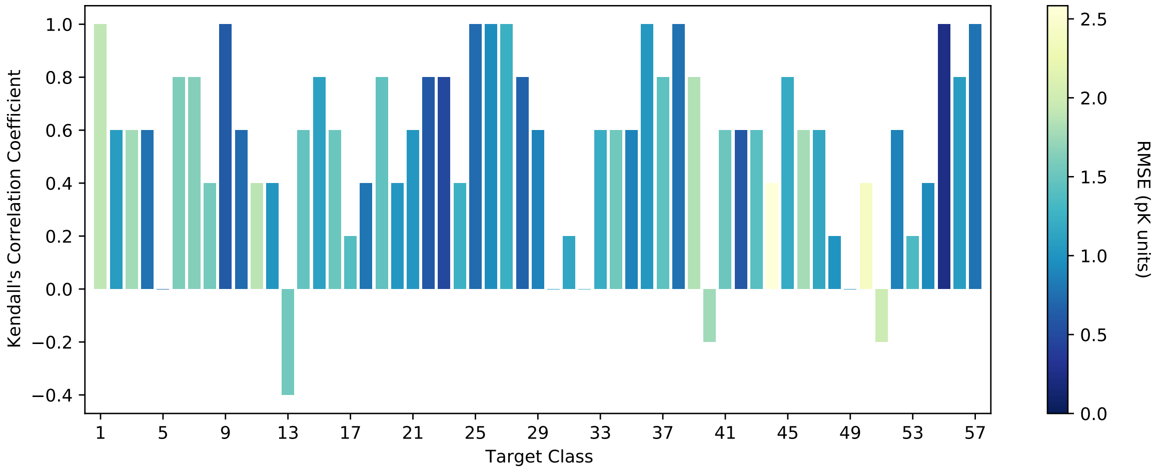

Figure 7: Per-class Kendall correlation coefficient, with each bar color-coded by the corresponding RMSE in pK units, for the 57 classes of the CASF-2016 dataset.

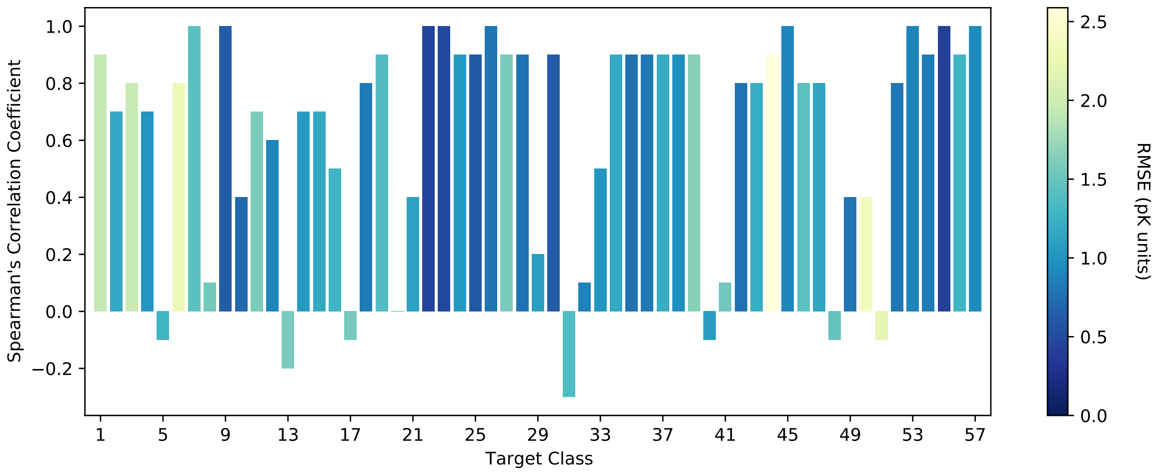

Figure 8: Per-class Spearman correlation coefficient, with each bar color-coded by the corresponding RMSE in pK units, for the 57 classes of the CASF-2016 dataset. The model is trained on systems without hydrogen atoms.

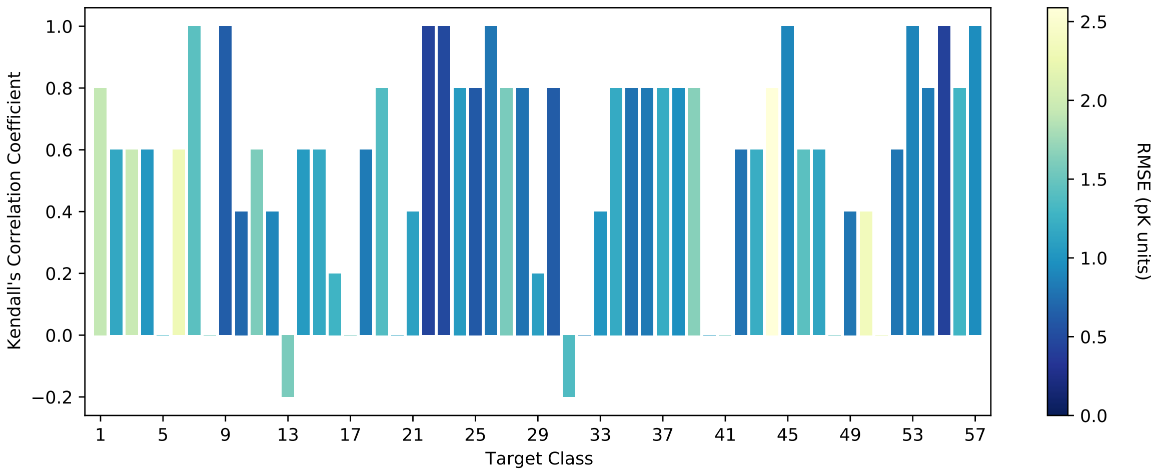

Figure 9: Per-class Kendall correlation coefficient, with each bar color-coded by the corresponding RMSE in pK units, for the 57 classes of the CASF-2016 dataset. The model is trained on systems without hydrogen atoms.

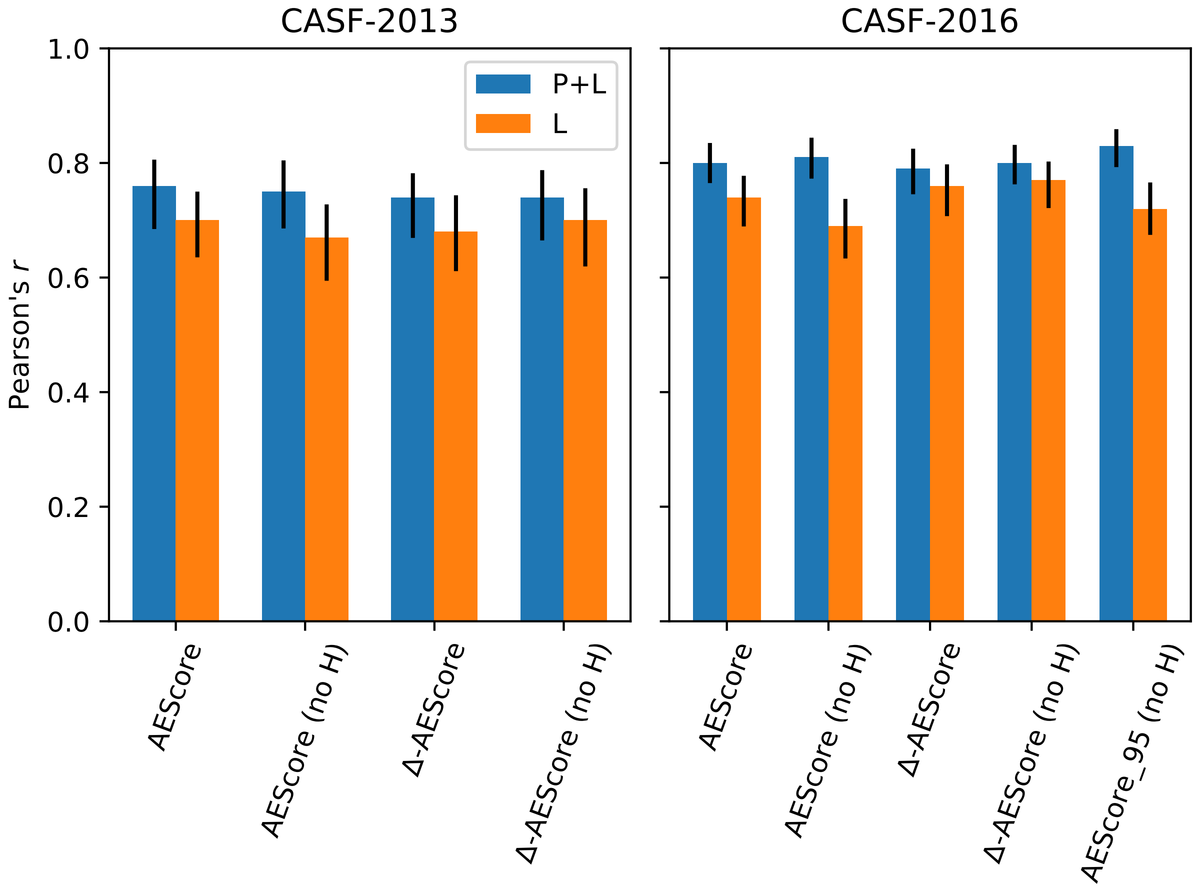

Figure 10: Pearson's correlation coefficient for different models incorporating atoms from the protein and the ligand (P + L, $d = 3.5$ Å) or atoms of the ligand only (L), for the CASF-2013 and CASF-2016 benchmarks, together with 90% confidence intervals.

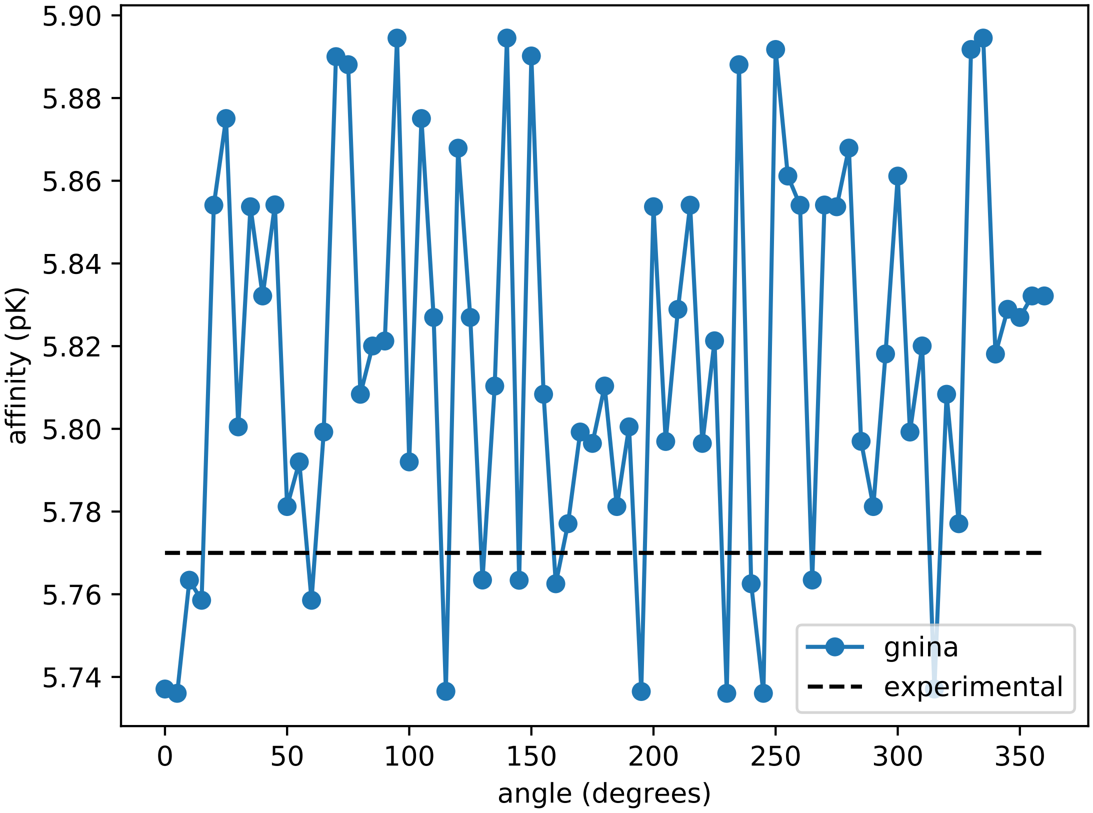

Figure 11: Predicted affinity as a function of the angle of rotation for the CNN-based scoring function `gnina`[3,4] on the 1O5B complex—the one with smaller absolute prediction error—of the CASF 2016 data set. Predictions are obtained using the pre-trained Default2018 model. The complex is rotated around the protein center of mass along the z axis.

Table 3: Comparison between models incorporating atoms from the protein and the ligand (P + L, $d = 3.5\,\text{Å}$) or atoms of the ligand only (L). The best performance for each test set is underlined. RMSE values are given in pK units.

Model	AEV	Test Set	RMSE	Pearson's r
AEScore	P + L	CASF-2013	<u>1.46</u>	<u>0.76</u>
AEScore	L	CASF-2013	1.65	0.70
AEScore (no H)	P + L	CASF-2013	1.48	0.75
AEScore (no H)	L	CASF-2013	1.69	0.67
Δ-AEScore	P + L	CASF-2013	1.53	0.74
Δ-AEScore	L	CASF-2013	1.65	0.68
Δ-AEScore (no H)	P + L	CASF-2013	1.52	0.74
Δ-AEScore (no H)	L	CASF-2013	1.61	0.70
Vina (optim)	—	CASF-2013	1.83	0.60
AEScore	P + L	CASF-2016	1.30	0.80
AEScore	L	CASF-2016	1.49	0.74
AEScore (no H)	P + L	CASF-2016	1.28	0.81
AEScore (no H)	L	CASF-2016	1.59	0.69
AEScore_{95} (no H)	P + L	CASF-2016	<u>1.22</u>	<u>0.83</u>
AEScore_{95} (no H)	L	CASF-2016	1.50	0.72
Δ-AEScore	P + L	CASF-2016	1.34	0.79
Δ-AEScore	L	CASF-2016	1.41	0.76
Δ-AEScore (no H)	P + L	CASF-2016	1.32	0.80
Δ-AEScore (no H)	L	CASF-2016	1.40	0.77
Vina (optim)	—	CASF-2016	1.75	0.60

Table 4: Performance of different machine learning and deep learning models for affinity prediction on the CASF-2013 and CASF-2016 benchmarks. NN denotes feed-forward neural networks, CNN denotes convolutional neural networks, and ML denotes "classical" machine learning methods (random forests, gradient boosting trees, ...). "Refined", "general" and "core" all refer to the PDBbind dataset.

Model	Type	Training Set	Test Set	RMSE	Pearson's r
AEScore†	NN	Refined 2016	CASF-2013	1.46	0.76
AEScore†(no H)	NN	Refined 2016	CASF-2013	1.48	0.75
RosENet[5]	CNN	Refined 2016	CASF-2013	1.43	0.80
AGL-Score[6]	ML	Refined 2016	CASF-2013	1.46	0.79
1D2D CNN[7]	CNN	Refined 2013	CASF-2013	1.47	0.78
Res4HTMD[5]	CNN	Refined 2016	CASF-2013	1.48	0.77
NNScore 2.0[8,9]*	ML	General 2018	CASF-2013	—	0.75
RF Score[9,10]*	ML	General 2018	CASF-2013	—	0.75
Pafnucy[11]	CNN	General 2016	CASF-2013	1.62	0.70
Vina (optim)	—	—	CASF-2013	1.82	0.61
AEScore†	NN	Refined 2016	CASF-2016	1.30	0.80
AEScore†(no H)	NN	Refined 2016	CASF-2016	1.28	0.81
AEScore$_{95}$†(no H)	NN	Refined 2016 - 95%	CASF-2016	1.22	0.83
AGL-Score[6]	ML	Refined 2016	CASF-2016	1.28	0.83
NNScore 2.0[8,9]*	NN	General 2018	CASF-2016	—	0.82
RF Score[9,10]*	ML	General 2016	CASF-2016	—	0.81
pair[12]	NN	Refined 2018	CASF-2016	1.45	0.78
Vina (optim)	—	—	CASF-2016	1.75	0.59
AEScore†	NN	Refined 2016	Core 2016	1.32	0.80
AEScore†(no H)	NN	Refined 2016	Core 2016	1.32	0.81
AEScore$_{95}$†(no H)	NN	Refined 2016 - 95%	Core 2016	1.22	0.83
1D2D CNN[7]	CNN	Refined 2016	Core 2016	1.21	0.85
Res4HTMD[5]	CNN	Refined 2016	Core 2016	1.25	0.83
RosENet[5]	CNN	Refined 2016	Core 2016	1.24	0.82
KDeep[13]	CNN	Refined 2016	Core 2016	1.27	0.82
AutoDock & RF-Score[14]	ML	Refined 2016	Core 2016	1.36	0.82
DeepAtom[15]	CNN	Refined 2016	Core 2016	1.32	0.81
AK-score (ensemble)[16]	CNN	Refined 2018	Core 2016	—	0.81
gnina[17,18]•	CNN	General 2016	Core 2016	1.37	0.80
RF Score[10,13]	ML	Refined 2016	Core 2016	1.39	0.80
Pafnucy[11]	CNN	General 2016	Core 2016	1.42	0.78
gnina[17,18]•	CNN	Refined 2016	Core 2016	1.50	0.73
AK-score single[16]	CNN	Refined 2018	Core 2016	—	0.76

† This work.

* Systems that could not be parsed by OpenBabel or RDKit were excluded from the test sets, resulting in 180 and 276 complexes for CASF-2013 and CASF-2016, respectively.

• 280 protein-ligand complexes in the test set.

References

(1) Gao, X.; Ramezanghorbani, F.; Isayev, O.; Smith, J. S.; Roitberg, A. E. TorchANI: A Free and Open Source PyTorch-Based Deep Learning Implementation of the ANI Neural Network Potentials. *J. Chem. Inf. Model.* **2020**, *60*, 3408–3415.

(2) Liu, Z.; Su, M.; Han, L.; Liu, J.; Yang, Q.; Li, Y.; Wang, R. Forging the Basis for Developing Protein–Ligand Interaction Scoring Functions. *Acc. Chem. Res.* **2017**, *50*, 302–309.

(3) Ragoza, M.; Turner, L.; Koes, D. R. Ligand Pose Optimization with Atomic Grid-Based Convolutional Neural Networks. 2017.

(4) McNutt, A. T.; Francoeur, P.; Aggarwal, R.; Masuda, T.; Meli, R.; Ragoza, M.; Sunseri, J.; Koes, D. R. GNINA 1.0: molecular docking with deep learning. *J. Cheminform.* **2021**, *13*, 43.

(5) Hassan-Harrirou, H.; Zhang, C.; Lemmin, T. RosENet: Improving Binding Affinity Prediction by Leveraging Molecular Mechanics Energies with an Ensemble of 3D Convolutional Neural Networks. *J. Chem. Inf. Model.* **2020**, *60*, 2791–2802.

(6) Nguyen, D. D.; Wei, G.-W. AGL-Score: Algebraic Graph Learning Score for Protein–Ligand Binding Scoring, Ranking, Docking, and Screening. *J. Chem. Inf. Model.* **2019**, *59*, 3291–3304.

(7) Cang, Z.; Mu, L.; Wei, G.-W. Representability of algebraic topology for biomolecules in machine learning based scoring and virtual screening. *PLoS Comput Biol* **2018**, *14*, e1005929.

(8) Durrant, J. D.; McCammon, J. A. NNScore 2.0: A Neural-Network Receptor–Ligand Scoring Function. *J. Chem. Inf. Model.* **2011**, *51*, 2897–2903.

(9) Boyles, F.; Deane, C. M.; Morris, G. M. Learning from the ligand: Using ligand-based features to improve binding affinity prediction. *Bioinformatics* **2019**, *36*, 758–764.

(10) Ballester, P. J.; Mitchell, J. B. A machine learning approach to predicting protein–ligand binding affinity with applications to molecular docking. *Bioinformatics* **2010**, *26*, 1169–1175.

(11) Stepniewska-Dziubinska, M. M.; Zielenkiewicz, P.; Siedlecki, P. Development and evaluation of a deep learning model for protein–ligand binding affinity prediction. *Bioinformatics* **2018**, *34*, 3666–3674.

(12) Zhu, F.; Zhang, X.; Allen, J. E.; Jones, D.; Lightstone, F. C. Binding Affinity Prediction by Pairwise Function Based on Neural Network. *J. Chem. Inf. Model.* **2020**, *60*, 2766–2772.

(13) Jiménez, J.; Škalič, M.; Martínez-Rosell, G.; De Fabritiis, G. KDEEP: Protein–ligand Absolute Binding Affinity Prediction via 3D-Convolutional Neural Networks. *J. Chem. Inf. Model.* **2018**, *58*, 287–296.

(14) Afifi, K.; Al-Sadek, A. F. Improving classical scoring functions using random forest: The non-additivity of free energy terms' contributions in binding. *Chem. Biol. Drug. Des.* **2018**, *92*, 1429–1434.

(15) Li, Y.; Rezaei, M. A.; Li, C.; Li, X.; Wu, D. DeepAtom: A Framework for Protein-Ligand Binding Affinity Prediction. 2019.

(16) Kwon, Y.; Shin, W.-H.; Ko, J.; Lee, J. AK-Score: Accurate Protein-Ligand Binding Affinity Prediction Using the Ensemble of 3D-Convolutional Neural Network. 2020.

(17) Ragoza, M.; Hochuli, J.; Idrobo, E.; Sunseri, J.; Koes, D. R. Protein–Ligand Scoring with Convolutional Neural Networks. *J. Chem. Inf. Model.* **2017**, *57*, 942–957.

(18) Francoeur, P. G.; Masuda, T.; Sunseri, J.; Jia, A.; Iovanisci, R. B.; Snyder, I.; Koes, D. R. Three-Dimensional Convolutional Neural Networks and a Cross-Docked Data Set for Structure-Based Drug Design. *J. Chem. Inf. Model.* **2020**, *60*, 4200–4215.
